# Implementation Science for the Prevention and Treatment of HIV among Adolescents and Young Adults in Sub-Saharan Africa: A Scoping Review

**DOI:** 10.1007/s10461-022-03770-x

**Published:** 2022-08-10

**Authors:** Susan Vorkoper, Kadija M. Tahlil, Nadia A. Sam-Agudu, Joseph D. Tucker, Alicia A. Livinski, Frances Fernando, Rachel Sturke

**Affiliations:** 1 Fogarty International Center, National Institutes of Health, Bethesda, MD USA; 2 Department of Epidemiology, Gillings School of Global Public Health, University of North Carolina at Chapel Hill, Chapel Hill, NC USA; 3 Pediatric &Adolescent HIV Unit and International Research Center of Excellence, Institute of Human Virology Nigeria, Abuja, Nigeria; 4 Institute of Human Virology, Department of Pediatrics, University of Maryland School of Medicine, Baltimore, MD USA; 5 Institute of Global Health and Infectious Diseases, University of North Carolina at Chapel Hill, Chapel Hill, NC USA; 6 Faculty of Infectious and Tropical Diseases, London School of Hygiene and Tropical Medicine, London, UK; 7 Office of Research Services, National Institutes of Health (NIH) Library, Bethesda, OD, NIH, MD USA; 8 Eunice Kennedy Shriver National Institute of Child Health and Human Development, Bethesda, MD USA

**Keywords:** Implementation science, HIV, Sub-saharan Africa, Adolescent, Young adult

## Abstract

Despite many evidence-based adolescent and young adult (AYA) HIV interventions, few are implemented at scale in sub-Saharan Africa (SSA). A growing implementation science literature provides important context for scaling up AYA HIV interventions in this high HIV-burden region. This scoping review examined the use of implementation research in AYA HIV studies conducted in SSA. We searched five databases and included articles which focused on AYA (10–24 years old), addressed HIV prevention or treatment, were conducted exclusively in SSA countries, and included an implementation science outcome. We included 44 articles in 13 SSA countries. Most were in East (52.3%) and South Africa (27.3%), and half focused exclusively on HIV prevention components of the care continuum. Acceptability and feasibility were the most cited implementation science outcomes. Only four articles used an established implementation science framework. The findings informed our recommendations to guide the design, implementation, and dissemination of further studies and health policymaking.

## Introduction

HIV is a leading cause of death for adolescents and young people (AYA) 10–24 years old in sub-Saharan Africa (SSA) [1, 2]. HIV eradication for any population depends heavily on the successful implementation of effective interventions that address their health and life context. Implementation science is the study of methods to promote the adoption and integration of evidence-based practices, interventions and policies into routine health care and public health settings [3]. Implementation science involves the use of “theories, models, and frameworks to optimize study design, data collection, analysis, and dissemination” [4]. This scientific approach enables researchers and program implementers focused on HIV among AYA to address barriers to implementing evidence-based health interventions, including lack of youth-friendly health services, gender-based violence and harmful cultural and social norms that limit access to, and uptake of, health services [5].

Despite the availability of proven health interventions for prevention and treatment, high rates of HIV infection continue to disproportionately affect AYA, especially in SSA [6]. It is clear that the promise of implementation science for addressing this continuing health problem has not been fully realized. The Adolescent HIV Prevention and Treatment Implementation Science Alliance (AHISA) was established by the Fogarty International Center in 2017 to address this challenge by creating a platform for bidirectional learning between researchers and users of research evidence [3, 7]. AHISA helps to facilitate better use of scientific evidence in adolescent HIV programming, while simultaneously supporting country-driven implementation research that is responsive to the local context [7].

Few reviews have focused on HIV implementation science among AYA in SSA. Available reviews of implementation science studies have focused on HIV prevention and treatment for adults and examined global, not SSA-specific data [8], or examined AYA engagement in SSA, but without a focus on implementation science [9]. This underlines the need for a synthesis of implementation science-guided adolescent HIV prevention and treatment research in SSA.

Therefore, this scoping review provides an overview of the published literature describing the extent, range, and nature of implementation science studies to address prevention and treatment of HIV among AYA in SSA and to discuss implications for future research, policies, and programs.

## Methods

The Preferred Reporting Items for Systematic Reviews and Meta-Analyses extension for Scoping Reviews (PRISMA-ScR) was used for reporting this review [10]. We selected a scoping review for the following reasons: heterogeneity of implementation science studies often does not sufficiently allow for pooling and standardized analysis of outcomes; substantial heterogeneity in key operational definitions; and identifying gaps in the literature may be better addressed through a scoping review [11].

### Eligibility criteria

We used the following inclusion criteria: addressed HIV prevention or treatment, included a study population of people between 10 and 24 years old, was conducted exclusively in SSA countries, an original research article, published since 2010 in the English language, and included the consideration and/or measurement of at least one stated implementation science outcome (specifically, acceptability, adoption, appropriateness, costs, feasibility, fidelity, penetration, sustainability, and scale-up) [12]. Articles with a study population (or study participants) outside of the 10–24 years old age range were excluded. Studies where caregivers, health professionals, teachers, or adults 25 years or older were engaged as part of an intervention targeting youth were included.

### Information sources and search strategy

In October 2020 and September 2021, a biomedical librarian searched five citation and abstract databases: Embase (Elsevier), Global Health (CAB Direct), PubMed (US National Library of Medicine), Scopus (Elsevier), and Web of Science: Core Collection (Clarivate Analytics). The biomedical librarian developed the search strategies using a combination of controlled vocabulary terms (i.e., Medical Subject Headings, EMTREE, CAB Direct thesaurus) and keywords for each concept of interest (AYA, HIV prevention and treatment, implementation science, SSA) recommended by subject matter experts and informed by reviewing key articles (see Supplemental File 1 for final search strategies used). The searches were limited to those published after 2010 and in the English language, and publication types other than original research and reviews were excluded. EndNote X9.3.3 (Clarivate Analytics) was used to collect and manage the retrieved citations and identify duplicates.

### Selection of sources

Covidence (Veritas Health Innovations, Ltd.), a systematic review web-based management platform, was used for screening. First, three reviewers (SV, KMT, FF) independently screened the titles and abstracts using the eligibility criteria. Those that had unknown or missing information were included for additional screening. Disagreements in screening were resolved by consensus between the three reviewers. However, as many of the abstracts did not indicate the specific age of the study populations, the full text of the articles was screened specifically for the ages of interest. Articles that included any age outside of 10–24 years old were excluded.

For those articles that met inclusion after both title and abstract screening and screening the full text with the age criterion, another screening of the full text was conducted by three reviewers (SV, KMT, RS) using the above-mentioned eligibility criteria. Disagreements were resolved by consensus between reviewers.

### Data charting

Data was extracted by the three reviewers (SV, KMT, RS) into Microsoft Excel using an established codebook (Supplemental File 2). We collected information from each article on: demographics (e.g., age, sex) of the population studied; country; whether an implementation framework or theory was used; implementation phase implementation outcome(s); study design; implementation strategy; and type of intervention. One reviewer (SV) screened all the data to identify discrepancies in the extracted information. These discrepancies were resolved by consensus between all three reviewers.

We also examined the extent of youth engagement for each study included, using the engagement typology developed by Asuquo et al. [9]. First, we defined youth engagement as working collaboratively with AYA who share common goals and interests through building authentic partnerships, which include mutual respect, inclusive participation, and equitable relationships. We classified youth engagement into four categories [9], including none, minimal, moderate, or substantial. Studies had no youth engagement when there was an absence of participatory approaches or activities during research; minimal youth engagement when youth were consulted to get their opinions, assigned specific roles, or informed about events surrounding research activities, but had no decision-making power; moderate youth engagement when there was adult-initiated activities with shared decision making between youth and adults; and substantial engagement when youth were included as co-researchers.

We reviewed the first and last author affiliations of each included article to try to understand issues of research capacity and of power-sharing in the allocation of authorship roles in the collaborative projects that produced the articles reviewed. Authorship position was selected as an indicator of research capacity under the assumption that the standard first and last author positions signify who did the majority of the manuscript writing, and who oversaw the design, implementation, and conduct of the study, respectively. For each article, we recorded in Excel all affiliations and countries for the first and last authors listed on each article. If an author listed more than one affiliation or different countries, we included all listed affiliations and countries. In cases where the country was not included in the affiliation list, we used Google to search and determine where each institution is located.

## Results

### Selection of sources of evidence

**Fig. 1:**
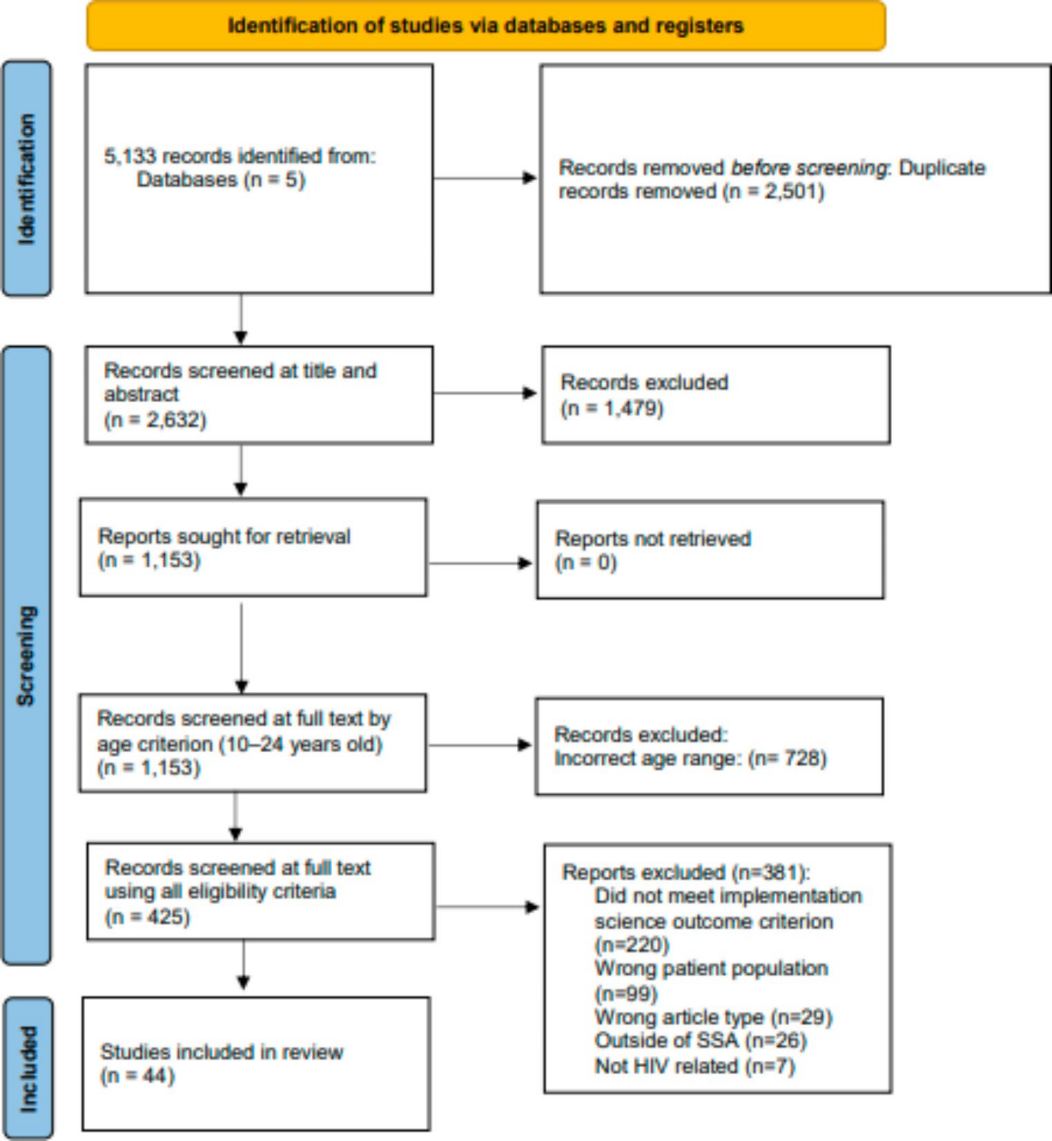
PRISMA Flow Diagram

A total of 5,133 citations were retrieved by the literature searches, of which 2,501 were duplicates and 2,632 were unique and screened. Of the 2,632 screened at the title and abstract level, 1,479 were excluded and 1,153 included. These 1,153 citations were next screened at the full text level by the age criterion (e.g., 10–24 years old only), and 728 were excluded as outside the age range. This resulted in 425 records screened at the full text level with the remaining eligibility criteria, of which 381 were excluded. A total of 44 articles were included at the end of the full text screening step (Fig. 1; Table 1).

**Table 1:** Summary of included HIV implementation science studies for adolescents in sub-Saharan Africa between 2010–2021 (n = 44)

Author (Year)	Country	Sample Size	AYA Partici-pants Age (Yrs.)	Adult Popula-tion	Study Design	Implementation			
						**Study Phase**	**Outcome**	**Strategies**	**Model or Framework**
Dunbar (2010)[13]	Zimbabwe	50	15–19	None	Observational	Describe implementation process	Feasibility	Engage consumers Utilize financial strategies Train and educate stakeholders Use evaluative and iterative strategies	None
Sayles (2010)[14]	South Africa	42	18–24	None	Observational	Pre-implementation	Acceptability	Engage consumers	Deductive Framework Approach
Rijsdijk (2011)[15]	Uganda	1864	12–19	None	Quasi-experimental	Test implementation strategy/strategies	Fidelity	Train and educate stakeholders	None
MacPhail (2013)[16]	South Africa	29	14–17	Caregivers (n = 29)	Experimental	Test implementation strategy/strategies	Acceptability Feasibility	Engage consumers Use evaluative and iterative strategies Train and educate stakeholders Utilize financial strategies	None
Parker (2013)(17)	Congo	13	15–24	None	Pre-post design	Test implementation strategy/strategies	Adoption	Use evaluative and iterative strategies Adapt and tailor to the context Train and educate stakeholders	Diffusion of Innovations ADAPT
Parker (2013)(18)	DRC	13	15–24	None	Observational	Describe implementation process Test implementation strategy/strategies	Acceptability Feasibility	Use evaluative and iterative strategies Train and educate stakeholders	None
St. Lawrence (2013)[19]	Botswana	111	13–19	None	Case Study	Pre-implementation	Adoption	Engage consumers Use evaluative and iterative strategies Adapt and tailor to the context Train and educate stakeholders	None
Jemmott (2014)[20]	South Africa	89	15–24	None	Experimental	Describe implementation process Test implementation strategy/strategies	Acceptability	Engage consumers Use evaluative and iterative strategies Adapt and tailor to the context Develop stakeholder interrelationships	None
Snyder (2014)(21)	South Africa	109	16–24	None	Observational	Describe implementation process Test implementation strategy/strategies	Acceptability Feasibility	Engage consumers Use evaluative and iterative strategies Adapt and tailor to the context	None
Ybarra (2014)(22)	Uganda	366	13–19	None	Experimental	Test implementation strategy/strategies	Acceptability Feasibility	Engage consumers Use evaluative and iterative strategies Train and educate stakeholders	None
Smith (2016)(23)	South Africa	224	16–24	None	Observational	Pre-implementation	Acceptability Feasibility	Engage consumers Train and educate stakeholders	None
Dietrich (2018)[24]	South Africa	50	21–24	None	Observational	Pre-implementation	Acceptability Feasibility	Use evaluative and iterative strategies Change infrastructure	None
Dow (2018)[25]	Tanzania	58	12–24	None	Experimental	Describe implementation process	Acceptability Feasibility	Engage consumers	None
Hector (2018)[26]	Mozambique	496	16–20	None	Quasi-experimental	Describe implementation process Test implementation strategy/strategies	Acceptability	Use evaluative and iterative strategies Train and educate stakeholders	None
James (2018)(27)	South Africa	15	15–19	Healthcare Providers (n = 4); Facility Managers (n = 4)	Observational	Describe implementation process	Acceptability Feasibility	Engage consumers Train and educate stakeholders	None
Nacken (2018)(28)	Tanzania	20	12–14	Teachers (n = 22)	Observational	Pre-implementation	Acceptability	Engage consumers Use evaluative and iterative strategies Develop stakeholder interrelationships Train and educate stakeholders	Precede-proceed
Barker (2019)(29)	Ghana	35	12–18	None	Observational	Describe implementation process Test implementation strategy/strategies	Acceptability Feasibility	Engage consumers Use evaluative and iterative strategies Train and educate stakeholders Develop stakeholder interrelationships Support clinicians	None
Bernays (2019)[30]	Uganda	33	10–24	None	Case Study	Describe implementation process Test implementation strategy/strategies	Acceptability	Engage consumers Use evaluative and iterative strategies	None
Carney (2019)(31)	South Africa	100	16–21	None	Experimental	Test implementation strategy/strategies	Acceptability Feasibility	Train and educate stakeholders Engage consumers	None
Donenberg (2019)(32)	Rwanda	14	14–24	None	Experimental	Describe implementation process Test implementation strategy/strategies	Acceptability Feasibility Fidelity	Use evaluative and iterative strategies Train and educate stakeholders	EPIS (Exploration, Preparation, Implementation, Sustainment)
Harding (2019)(33)	Tanzania	48	14–18	None	Experimental	Describe implementation process Test implementation strategy/strategies	Acceptability Feasibility	Engage consumers Use evaluative and iterative strategies Train and educate stakeholders	None
Kibel (2019)[34]	Kenya	116	12–24	None	Quasi-experimental	Describe implementation process Test implementation strategy/strategies	Acceptability	Engage consumers Use evaluative and iterative strategies Adapt and tailor to the context	None
Mavhu (2019)[35]	Zimbabwe	618	13–17	None	Observational	Describe implementation process	Acceptability	None	None
Sabben (2019)[36]	Kenya	60	11–14	Parents (n = 22)	Experimental	Describe implementation process Test implementation strategy/strategies	Acceptability Feasibility	Change infrastructure Train and educate stakeholders Use evaluative and iterative strategies Adapt and tailor to the context	None
Smith (2019)[37]	South Africa	303	16–24	None	Observational	Pre-implementation	Acceptability	Engage consumers	None
Tonen-Wolyec (2019)(38)	DRC	628	15–19	Peer Educators (n = 36)	Observational	Describe implementation process Test implementation strategy/strategies	Acceptability Feasibility	Provide interactive assistance Train and educate stakeholders	None
Wogrin (2019)[39]	Zimbabwe	10	18–21	Peer Counsellors (n = 10)	Observational	Describe implementation process	Acceptability Feasibility	Engage consumers Use evaluative and iterative strategies Train and educate stakeholders	None
Dow (2020)[40]	Tanzania	128	12–24	None	Experimental	Describe implementation process Test implementation strategy/strategies	Acceptability Feasibility	Engage consumers Use evaluative and iterative strategies Train and educate stakeholders Develop stakeholder interrelationships	None
Dulli (2020)[41]	Nigeria	324	15–24	None	Experimental	Describe implementation process Test implementation strategy/strategies	Acceptability	Engage consumers Use evaluative and iterative strategies Adapt and tailor to the context Develop stakeholder interrelationships	None
Gill (2020)[42]	South Africa	148	15–19	None	Observational	Describe implementation process Test implementation strategy/strategies	Acceptability	Engage consumers	None
Iwelunmor (2020)[43]	Nigeria	903	10–24	None	Observational	Pre-implementation	Feasibility	Engage consumers Develop stakeholder interrelationships	None
Kidman (2020)(44)	Tanzania	2191	14–19	None	Observational	Describe implementation process	Acceptability Appropriateness	Engage consumers Use evaluative and iterative strategies	None
Kuo (2020)[45]	South Africa	196	13–15	Parents (n = 98)	Experimental	Describe implementation process	Acceptability Feasibility Fidelity	Provide interactive assistance Train and educate stakeholders Engage consumers	None
MacCarthy (2020)[46]	Uganda	179	15–24	Providers (n = 7)	Experimental	Test implementation strategy/strategies	Acceptability Feasibility	Engage consumers Use evaluative and iterative strategies Train and educate stakeholders	None
Nalukwago (2020)(47)	Uganda	Not reported	15–24	None	Observational	Test implementation strategy/strategies	Fidelity	Use evaluative and iterative strategies Adapt and tailor to the context Provide interactive assistance Engage consumers	Diffusion of Innovations
Abiodun (2021)[48]	Nigeria	209	15–19	None	Experimental	Describe implementation process Test implementation strategy/strategies	Acceptability Feasibility	Train and educate stakeholders Engage consumers	None
Catania (2021)[49]	Tanzania	257	14–19	None	Experimental	Describe implementation process Test implementation strategy/strategies	Fidelity	Engage consumers Use evaluative and iterative strategies Train and educate stakeholders	None
Chory (2021)[50]	Kenya	30	10–19	None	Mixed Methods	Describe implementation process Test implementation strategy/strategies	Acceptability Feasibility	Engage consumers Use evaluative and iterative strategies Adapt and tailor to the context	None
Colombini (2021)[51]	Tanzania South Africa	563	16–24	Clinical Staff (n = 13)	Quasi-experimental	Describe implementation process Test implementation strategy/strategies	Acceptability Feasibility Fidelity	Use evaluative and iterative strategies Provide interactive assistance	None
Koris (2021)[52]	Zimbabwe	52	18–24	None	Case Study	Describe implementation process Test implementation strategy/strategies	Acceptability	Engage consumers Use evaluative and iterative strategies	None
Mulwa (2021)[53]	Kenya	606	10–24	None	Quasi-experimental	Describe implementation process Test implementation strategy/strategies	Feasibility	Engage consumers Use evaluative and iterative strategies	None
Stangl (2021)[54]	Zambia	24	15–19	None	Mixed Methods	Describe implementation process Test implementation strategy/strategies	Acceptability Feasibility	Engage consumers Use evaluative and iterative strategies Adapt and tailor to the context Train and educate stakeholders	None
Tahlil (2021)[55]	Nigeria	42	14–24	None	Observational	Pre-implementation	Feasibility	Engage consumers Use evaluative and iterative strategies Adapt and tailor to the context Develop stakeholder interrelationships Train and educate stakeholders	None
Ybarra (2021)(56)	Uganda	202	18–22	None	Experimental	Describe implementation process Test implementation strategy/strategies	Acceptability Feasibility	Engage consumers Use evaluative and iterative strategies Adapt and tailor to the context	None

### Descriptive characteristics

**Fig. 2:**
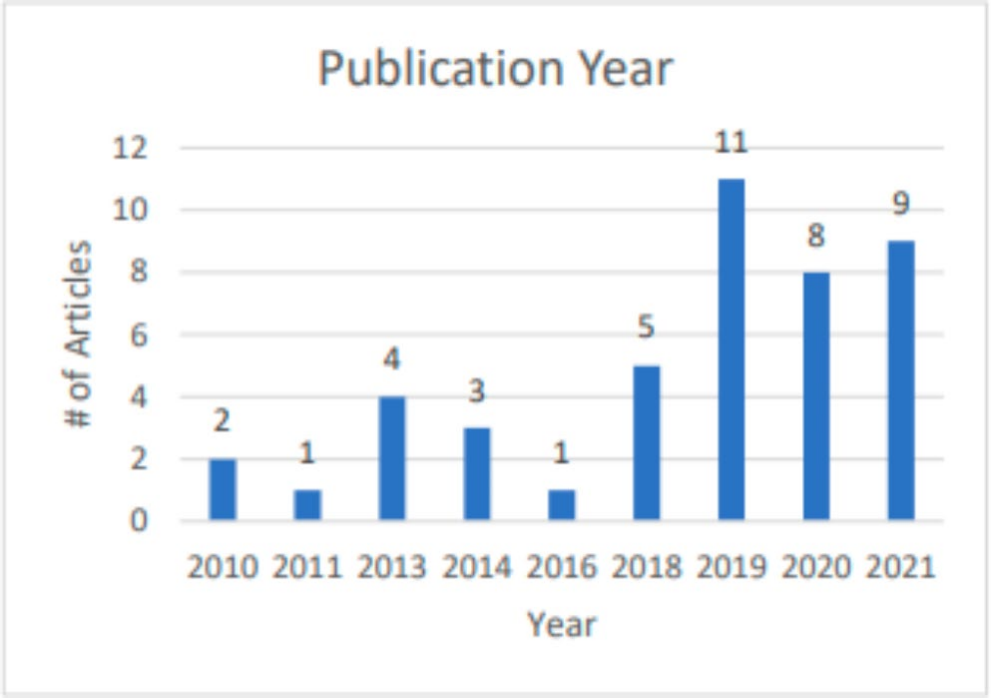
Number of Publications by Year

Table 2 shows the descriptive characteristics of the 44 included articles. Included studies took place in 13 SSA countries. The largest share of the studies (52.3%) focused on the East African region; 27.3% (12/44) focused on the southern African region. South Africa alone was the setting for 25.0% of all studies. The West (11.4%) and Central (6.8%) African regions were less well represented. Three quarters (75.0%) of the studies were published in the last four years, eclipsing the previous six years, which accounted for only 11 (25%) of the 44 articles (Fig. 2). It is worth noting that all of the studies published in 2021 took place outside of South Africa and had geographic diversity across the East, West, and South regions.

**Table 2:** Characteristics of adolescent HIV implementation science studies in sub-Saharan Africa between 2010–2021 (n = 44)

	n # of studies	% of total studies
**United Nations sub-Saharan Regions**		
Central	3	6.8
East	23	52.3
South	12	27.3
West	5	11.4
Multi-region (South & East)	1	2.3
**Gender of participants**		
Female only	8	18.2
Male only	2	4.5
Transgender	0	0.0
Male and female	29	65.9
Not specified	5	11.4
**Age**		
10—14 years old	3	6.8
15—19 years old	5	11.4
20—24 years old	1	2.3
Across all ages (10–24 years old)	35	79.5
**Participants’ HIV Status**		
Positive only	15	34.1
Negative only	2	4.5
Both	9	20.5
Not specified	18	40.9
**HIV Prevention, Care, and Treatment Continuum**		
Prevention	27	61.4
Care	0	0.0
Treatment	3	6.8
Across the continuum	14	31.8
**Intervention Delivery**		
Face-to-face only	31	70.5
Digital^a^ only	8	18.2
Face-to-face and digital	5	11.4
**Extent of Youth Engagement** ^**b**^		
None	27	61.4
Minimal	14	31.8
Moderate	1	2.3
Substantial	2	4.4

^*a*^ requiring the use of a mobile phone or internet connection

^*b*^ Categories as described by Asuquo et al. [9]

The majority of studies (79.5%) focused on the broader AYA age range (10–24 years old). While more than half (52.3%) included older AYA (15–24 years old), only three studies focused on younger adolescents (10–14 years old).

While 18 studies (40.9%) did not indicate HIV status, more than a third (n = 15, 34.1%) included people living with HIV and a fifth included both people living with and without HIV (n = 9, 20.5%). Males and females were both enrolled in 29 (65.9%) of the studies; eight studies (18.2%) had only female participants.

Studies cut across the HIV prevention (e.g., testing and PrEP), care (e.g., peer support, psychosocial support, and counseling), and treatment (e.g., anti-retroviral treatment initiation and viral suppression) continuum. Approximately one third (31.8%) of the studies addressed more than one component of the HIV continuum. More than half (61.4%) of the studies exclusively focused on prevention and only 6.8% focused exclusively on care.

### Implementation science characteristics

Articles included in this scoping review had to specify an implementation science outcome [12] with either a stated definition or defined measurement. Acceptability and feasibility were both measured in almost half (43.2%) of the articles (Table 3). Acceptability (25.8%) was the single most defined and measured implementation outcome within the set of studies. Other implementation science outcomes (i.e., feasibility, fidelity, and adoption) were measured or defined by only nine articles, and cost and scale-up/sustainability were not cited in any of the studies.

**Table 3:** Implementation Science Outcomes Measured in Reviewed Studies

Implementation Science Outcome	Summary Definition ^a^	n # of studies	% of total studies
**Acceptability & Feasibility**	-	19	43.2
**Acceptability**	Perception among stakeholders that intervention or innovation is agreeable.	11	25.0
**Feasibility**	Extent to which an intervention or innovation can be successfully used or carried out within a given setting.	4	9.1
**Fidelity**	Degree to which an intervention was implemented as prescribed or intended.	3	6.8
**Adoption**	Uptake; Intention or action to employ an innovation or evidence-based practice.	2	4.5
**More than one** ^**b**^	-	5	11.4

^*a*^ *From Proctor et al., 2011*

^*b*^*Acceptability, Feasibility & Fidelity* [3]; *Acceptability & Appropriateness* [1]; *Acceptability & Fidelity* [1]. *Note: Reach, Costs, Sustainability outcomes were not measured in these studies*

Measures of implementation science outcomes included both qualitative and quantitative assessments. Some, including Ybarra, 2014 [22], and Smith, 2016 [23], developed questionnaires using Likert scales to assess outcomes of acceptability and feasibility. Others, including Barker, 2019 [29] and James, 2018 [27], conducted interviews and focus groups with participants using semi-structured interview guides. Secondary data and process data noting things like consent, continued attendance, and referral requests for additional resources were also documented as implementation science outcomes, particularly acceptability [31, 33, 38]. Many of the studies, particularly those that included more than one implementation science outcome, employed both qualitative and quantitative. Overall, studies included in the review defined and developed outcome measures differently, and there was no consensus on the measurements across the articles.

The implementation phases identified by this review were pre-implementation, testing of implementation strategies, and describing the implementation process. None of the studies were designed or explicitly described as hybrid Types I, II, or III [57] nor were they focused on sustainability or the development of implementation science measures as part of the protocol. Eight of the articles were focused on the pre-implementation phase as defined by addressing efficacy, effectiveness and/or cost-effectiveness of an intervention or adapting an intervention [58]. Studies testing implementation strategies and describing the implementation process (including identifying barriers and facilitators) comprised 15.9% and 11.4% of the articles, respectively. However, these two implementation phases were described together in 54.5% of the studies. With respect to study design, the majority of studies utilized observational (40.9%) and experimental (24.1%) designs. Quasi-experimental (11.4%), case studies (6.8%), mixed methods (4.5%), and pre-post (2.3%) designs were also employed. Of the 44 articles, only four (9.0%) used an established implementation science framework: Parker et al. [17] used the Systematic Approach for Adapting Evidence-Based Behavioral Interventions Guidelines (ADAPT) framework to describe the process of implementing a United States-developed, evidence-based intervention for positive risk reduction at a pediatric hospital in the Democratic Republic of the Congo. The intervention focused on older adolescents (15 to 24 years) living with HIV, and the study documented adaptation of the intervention within a low-resource context. Nalukwago et al. [47] conducted a process evaluation using the Diffusion of Innovations framework to collect data on HIV program reach and factors influencing implementation of a health-communication intervention to reduce HIV infections among adolescents 15 to 19 years old in Uganda. Nacken et al. [28] structured a needs assessment using the PRECEDE component of the PRECEDE– PROCEED model to understand the needs of students and teachers in Tanzania regarding an HIV prevention intervention in primary schools. Donenberg et al. [32] applied the Exploration, Preparation, Implementation and Sustainment (EPIS) framework to a two-arm randomized group-controlled trial of Trauma-Informed Adherence-Enhanced cognitive behavioral therapy delivered by Rwandan youth leaders to adolescents living with HIV.

### Youth Engagement

Despite the focus on mostly older adolescents, there was little youth engagement across studies. Nearly two-thirds (61.4%) of studies had no youth engagement and 14 (31.8%) had minimal youth engagement. One study had moderate youth engagement, and two articles described substantially engaged youth as co-researchers.

### Use of Digital Technologies

One increasingly common way to deliver implementation science interventions to youth is through digital approaches, which include mobile health (mHealth) or internet-based electronic health (eHealth) innovations [59]. Thirteen of the 44 studies incorporated digital strategies (Table 4). All but two of these studies took place between 2018 and 2021, and five of the digital interventions focused on AYA living with HIV. These specific interventions ranged from passive use of technology (i.e., sending simple direct text reminder messages to youth participants) to soliciting active youth participation through smartphone games and social media support groups.

**Table 4:** Summary of AYA HIV implementation science studies that used digital approaches to deliver interventions (n = 13)

Year	Author	Country	Study Population	Intervention Delivery Description
2011	Rijsdijk	Uganda	Secondary school students	Comprehensive, low-tech, computer-based, interactive sex education program
2014	Ybarra	Uganda	Secondary school students	Five-lesson internet-based, comprehensive sexuality education program
2018	Dietrich	South Africa	Adolescent women	Mobile phone questionnaires designed to collect data on sexual risk behavior
2019	Bernays	Uganda	Adolescents living with HIV	Use of the audio diary method (audio recorder) to collect data on HIV treatment adherence behavior and adolescents’ potential acceptability of an intervention developed to encourage their adherence
2019	Sabben	Kenya	Adolescents in western Kenya	Narrative-based smartphone game designed to help prevent HIV
2020	Dulli	Nigeria	Adolescents living with HIV	Structured social media-based (private Facebook groups) support group intervention to encourage HIV treatment retention
2020	Iwelunmor	Nigeria	Adolescents in Nigeria	Adolescents with ideas on how to enhance uptake of HIV self-testing among Nigerian youth could submit their ideas to the study team through Google Forms, WhatsApp or email.
2020	MacCarthy	Uganda	Adolescents in Kampala	A mobile technology-based intervention informed by behavioral economics to increase treatment adherence
2021	Abiodun	Nigeria	Adolescents living with HIV in southwest Nigeria	An interactive and tailored short message services (SMS) intervention that sends treatment adherence reminders to adolescents living with HIV.
2021	Catania	Tanzania	Adolescents in Tanzania	Development of a potentially generalizable video graphic instruction book that trains adolescents on how to perform an HIV oral self-implemented test and encourage linkage-to-care.
2021	Chory	Kenya	Adolescents living with HIV	Mobile-based individual counseling and peer support services delivered through WhatsApp
2021	Tahlil	Nigeria	Adolescents in Nigeria	Adolescents participating in a crowdsourcing event to improve HIV testing among Nigerian youth developed HIV prevention interventions that utilized mobile applications, websites, and social media to deliver health services.
2021	Ybarra	Uganda	Older adolescents in Uganda	A comprehensive, theory-driven text messaging-based HIV prevention intervention that delivered HIV information to older adolescents

### Lead author Affiliation

**Fig. 3:**
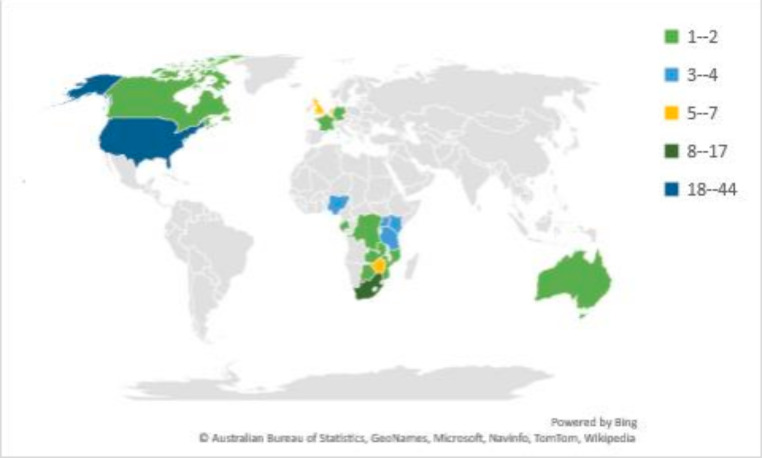
Countries of Institutional Affiliation Indicated by First and Last Authors (n = 107 institutions) *Multiple countries for all institutional affiliations of authors are included.

A review of the lead (first and last) authors’ stated affiliations (n = 107 affiliations among 95 first and last authors including co-first authors) showed that 58.9% [63] were employed by institutions outside SSA, with the majority of those (44, 69.8%) from the United States (Fig. 3). For institutions within SSA, which accounted for 41.1% [44] of total author affiliations, South Africa singularly represented 38.6% [17] of these affiliations. The East African region represented 47.7% [21] affiliations, and West Africa and Central Africa represented 11.4% [5] and 2.3% [1], respectively.

## Discussion

In this scoping review, HIV implementation science studies among AYA in SSA identified were largely conducted in Eastern and Southern Africa, with South Africa as the single most-represented country. While data from 2021 shows greater geographic diversity, Western and Central Africa were generally underrepresented in the overall group of studies reviewed. This has been the prevailing geographic trend in the broader HIV research landscape in SSA. A recent bibliographic analysis of HIV research in SSA found that East and Southern Africa generated approximately five times the amount of research compared to West and Central Africa, with Southern Africa accounting for a disproportionately large share [60].

We also found large discrepancies across age groups. While older adolescents and young adults aged 15 to 24 years comprised more than half of the study populations, young adolescents 10–14 years accounted for less than 10%. However, it is unclear if this is due to their being neglected in HIV studies, or whether this age group tends to be included in pediatric studies with children under 10 years of age.

Omission of participant HIV status in many of the studies is also notable. Some articles [56] acknowledged that they did not include this information in their criteria or their reporting. Others [28] were qualitative studies that may have refrained from requesting HIV status information in order to reduce perceived or experienced stigma and unintentional disclosure to other study participants.

There appeared to be a stronger focus on HIV prevention compared to the HIV care and treatment components in the HIV prevention, care, and treatment continuum of care. In contrast, a recent all age-inclusive systematic review of the HIV care cascade in SSA found that viral suppression was the most frequently studied component, followed by diagnosis and linkage to care [61]. This systematic review also included articles from as far back as 2004 and was not limited to implementation science studies, which may, along with the all age-inclusive focus, account for the contrasting findings. Our results may also indicate a shift in AYA-specific focus towards HIV prevention strategies such as PrEP and male circumcision, while striving to better understand implementation components related to uptake of these interventions.

Studies tended to focus more on the implementation outcomes of acceptability and feasibility, and none of the studies addressed penetration, cost, or sustainability and scale-up. This may be a matter of the availability of measurements and to what extent studies present and define their measures in the literature. A review of implementation outcome measurements reported that there were 50 acceptability instruments, compared to only eight on sustainability; the authors offered the explanation that acceptability has been a part of treatment and empirical research, while constructs like sustainability are new with respect to evidence-based practices [62]. Nwaozuru’s 2021 [63] review of mobile health technologies for AYA HIV in low- and middle-income countries found similar results. Additionally, acceptability and feasibility tend to be applied more at early-stage implementation, as they focus on ensuring that an intervention will be palatable to the populations of interest. In contrast, sustainability and scale-up are generally addressed once there has been uptake, and potentially, demonstrated effectiveness of an intervention. Therefore, the relatively high frequency of acceptability and feasibility measurements may suggest that researchers are more likely to think about implementation science metrics early in the research process. This is a possible indication of where the HIV implementation science field is, in terms of its application to AYA-targeted interventions in SSA.

Only four of included articles referenced a specific implementation science framework, specifically, ADAPT [18], Diffusion of Innovations [47], PRECEDE– PROCEED [28], and EPIS [32]. Neither the Consolidated Framework for Implementation Research (CFIR) nor the RE-AIM framework, two of the mostly widely used implementation science frameworks, were cited [64–66]. Implementation science frameworks are important tools for guiding research that is “intended to enhance the generalizability of findings by establishing common concepts and terminologies that can be applied across disparate research studies and settings” [65]. The low inclusion of frameworks in our review further reflects the nascent use of implementation science in the AYA HIV field in SSA. This calls for increasing capacity building and mentoring opportunities, including short- and long-term training programs in implementation science and mentored research. Furthermore, this finding infers a need to develop and adapt models and frameworks that are appropriate for geographical settings beyond the high-income country settings in which the majority of implementation science frameworks are developed.

We also found relatively low levels of youth engagement, which is consistent with a recent review [9] that reported that only 12% of the AYA HIV intervention studies conducted in SSA had substantial or moderate levels of youth engagement. Given the increasing use of and potential for mHealth technologies and virtual platforms in HIV research collaborations [67, 68], there are increasingly more options available for meaningfully engaging youth. Youth involvement in AYA-focused implementation science projects will be essential for developing and implementing acceptable, effective and sustainable interventions.

Almost 60% of studies reviewed had lead authors with affiliations outside of SSA. Hedt-Gauthier et al.’s 2019 [69] review of collaborative health research in Africa also reported a similar finding: 68% of articles had an author affiliated outside of the African country in which the study was conducted. This underscores the need for building implementation research capacity in SSA and the conduct of African-led implementation research, particularly in West and Central Africa, which accounted for less than 20% of the studies and only 13.6% of SSA authors in our study. One way to address and promote more African-led research is to create more research funding opportunities limited to local African applicants and investing in local resources, institutions, researchers, and training programs to design and conduct implementation science research across Africa. An additional approach is to include, integrate, and implement power-sharing approaches to scientific authorship in high-income and low-and middle-income countries collaborations [70, 71], in addition to similar considerations for study planning, design, and implementation.

This article has limitations that should be noted. First, we limited implementation science outcomes to those that were specifically labelled and defined, which may have eliminated potentially relevant articles that used slightly different language. We also had a strict age range of 10–24 years old and did not include any articles where the primary study populations fell outside of this range. It should be noted that we only included studies published in English; so French, Portuguese, or other relevant language papers were not reviewed.

Our data have implications for policymakers and researchers. From a policy perspective, our data highlight the need for more African-led AYA research studies. Given that global health funders increasingly see the value of African-led science, this may help to facilitate greater numbers of studies with first authors in SSA. Local fundraising and crowdfunding could support more African-led research studies [72]. From a research perspective, the data suggest the need for more implementation research focused on West and Central Africa. From the public health perspective, the continued high burden of HIV among AYA across all SSA regions warrants an expansion and maturation of implementation science in this topic area.

## Conclusions

The findings from this scoping review map out an exciting and important trajectory of research on AYA-focused implementation science across the HIV prevention, care, and treatment care continuums. Implementation science knowledge, practice, and scale up will help to better translate evidence-based interventions into practice and for the health and wellbeing of AYA in SSA.

## Electronic supplementary material

Below is the link to the electronic supplementary material.

Supplementary Material 1

Supplementary Material 2
