# Supplementary material for: Implementation Science for the Prevention and Treatment of HIV among Adolescents and Young Adults in Sub-Saharan Africa: A Scoping Review
Source: AIDS Behav. 2022 Aug 10;27(Suppl 1):7–23. doi: 10.1007/s10461-022-03770-x (PMC10191963; doi:10.1007/s10461-022-03770-x)
Supplement: Supplementary file 1 — Supplementary Material 1 [file 10461_2022_3770_MOESM1_ESM.docx]

Supplemental File 1. Final Search Strategies Used

**Database:** PubMed/MEDLINE
**Vendor:** US National Library of Medicine

**Date of Search:** October 28, 2020 & September 15, 2021
**Limits:** Language: English; Publication date: 1/1/2010 – 12/31/2021

**Notes:** Use Advanced search. Limit to title and abstract fields, title field, and MeSH fields for terms. Run main search first and then apply the limits to those results. Use the search strategy to exclude publication types of no interest.

**With BROAD Implementation science search strategy**

(Adolescent[mesh] OR adolescent[tiab] OR adolescents[tiab] OR adolescence[tiab] OR teenager[tiab] OR teenagers[tiab] OR teen[tiab] OR teens[tiab] OR youth[tiab] OR youths[tiab] OR “young adult”[tiab] OR “young adults”[tiab] OR “Young Adult”[mesh]) AND ((prevention[tiab] OR prevent*[tiab] OR preventative[tiab] OR screen*[tiab] OR screening*[tiab] OR treatment*[tiab] OR treat*[tiab] OR care[tiab] OR therapy[tiab] OR therapies[tiab] OR therapeutic*[tiab] OR "Preventive Health Services"[Mesh] OR "Primary Prevention"[Mesh] OR "Secondary Prevention"[Mesh] OR "Tertiary Prevention"[Mesh] OR "prevention and control"[Subheading] OR "Therapeutics"[Mesh] OR "therapy" [Subheading]) AND (HIV[tiab] OR hiv1[tiab] OR hiv2[tiab] OR hiv-1*[tiab] OR hiv-2*[tiab] OR HIV[mesh] OR AIDS[tiab] OR “human immunodeficiency virus”[tiab] OR “human immunodeficiency viruses”[tiab] OR “human immunedeficiency virus”[tiab] OR “human immuno-deficiency virus”[tiab] OR “acquired immunodeficiency syndrome”[tiab] OR “acquired immunodeficiency syndromes”[tiab] OR “acquired immunedeficiency syndrome”[tiab] OR “acquired immuno-deficiency syndrome”[tiab] OR “acquired immuno-deficiency syndromes”[tiab] OR “acquired immune-deficiency syndrome”[tiab] OR “acquired immune-deficiency syndromes”[tiab] OR “HIV Infections”[majr])) AND (implementing[ti] OR implementation[ti] OR “implementation theory”[tiab] OR “implementation theories”[tiab] OR "implementation science"[tiab] OR "implementation research"[tiab] OR “implementation intervention”[tiab] OR “implementation interventions”[tiab] OR "implementation strategy"[tiab] OR "implementation strategies"[tiab] OR “implementation framework”[tiab] OR “implementation frameworks”[tiab] OR “implementation barrier”[tiab] OR “implementation barriers”[tiab] OR “intervention research”[tiab] OR “intervention oriented research”[tiab] OR “improvement science”[tiab] OR "dissemination research"[tiab] OR "dissemination science"[tiab] OR "translational research"[tiab] OR "diffusion of innovation"[tiab] OR "impact evaluation"[tiab] OR “impact evaluations”[tiab] OR “impact research”[tiab] OR “knowledge transfer”[tiab] OR “transfer knowledge”[tiab] OR “knowledge translation”[tiab] OR “knowledge exchange”[tiab] OR “knowledge to action”[tiab] OR acceptability[tiab] OR adoption[tiab] OR appropriateness[tiab] OR feasibility[tiab] OR fidelity[tiab] OR sustainability[tiab] OR "implementation cost"[tiab] OR "implementation costs"[tiab] OR "implementation outcome"[tiab] OR "implementation outcomes"[tiab] OR "implementation process"[tiab] OR "implementation processes"[tiab] OR "effectiveness-implementation hybrid"[tiab] OR “community based implementation”[tiab] OR "ecological framework"[tiab] OR "ecological frameworks"[tiab] OR (“organizational readiness”[tiab] AND change[tiab]) OR "Re-AIM"[tiab] OR "reach effectiveness adoption implementation maintenance"[tiab] OR PARIHS[tiab] OR "promoting action on research implementation in health services"[tiab] OR CFIR[tiab] OR "PRECEDE-PROCEED"[tiab] OR “interactive systems framework*”[tiab] OR “Theoretical Domains Framework*”[tiab] OR AIF[tiab] OR “Active Implementation Framework*”[tiab] OR “Consolidated Framework for Implementation Research”[tiab] OR NICS[tiab] OR PRISM[tiab] OR SURE[tiab] OR TDF[tiab] OR “Theoretical Domains Framework*”[tiab] OR TICD[tiab] OR “Exploration Preparation Implementation Sustainment framework”[tiab] OR EPIS[tiab] OR "Diffusion of Innovation"[Mesh] OR "Implementation Science"[Mesh] OR "Health Plan Implementation"[Mesh]) AND (angola[MeSH] OR angola[tiab] OR benin[tiab] OR benin[mesh] OR botswana[tiab] OR Botswana[mesh] OR "Burkina faso"[mesh] OR "burkina faso"[tiab] OR burundi[MeSH] OR burundi[tiab] OR "cabo verde"[tiab] OR cameroon[MeSH] OR cameroon[tiab] OR "central African republic"[tiab] OR "central African republic"[mesh] OR chad[tiab] OR chad[mesh] OR comoros[tiab] OR comoros[mesh] OR "Cote d'Ivoire"[mesh] OR "Cote d'Ivoire"[tiab] OR "Ivory Coast"[tiab] OR "Democratic Republic of the Congo"[tiab] OR congo[MeSH] OR congo[tiab] OR "republic of the congo"[tiab] OR "equatorial guinea"[tiab] OR "equatorial guinea"[mesh] OR Eritrea[mesh] OR Eritrea[tiab] OR Eswatini[tiab] OR Ethiopia[tiab] OR Ethiopia[mesh] OR gabon[tiab] OR gabon[mesh] OR gambia[mesh] OR gambia[tiab] OR ghana[MeSH] OR ghana[tiab] OR guinea[tiab] OR guinea[mesh] OR "guinea-bissau"[tiab] OR "guinea-bissau"[mesh] OR kenya[MeSH] OR kenya[tiab] OR lesotho[MeSH] OR lesotho[tiab] OR Liberia[tiab] OR Liberia[mesh] OR Madagascar[tiab] OR Madagascar[mesh] OR malawi[MeSH] OR malawi[tiab] OR mali[tiab] OR mali[mesh] OR Mauritania[mesh] OR Mauritania[tiab] OR Mauritius[mesh] OR Mauritius[tiab] OR mozambique[MeSH] OR mozambique[tiab] OR namibia[MeSH] OR namibia[tiab] OR niger[tiab] OR niger[mesh] OR nigeria[MeSH] OR nigeria[tiab] OR rwanda[MeSH] OR rwanda[tiab] OR "sao tome and principe"[tiab] OR "sao tome"[tiab] OR "sao tome and principe"[mesh] OR Senegal[tiab] OR Senegal[mesh] OR Seychelles[tiab] OR Seychelles[mesh] OR "sierra leone"[tiab] OR "sierra leone"[mesh] OR Somalia[tiab] OR Somalia[mesh] OR Somaliland[tiab] OR "South Africa"[tiab] OR "south Africa"[mesh] OR "south sudan"[mesh] OR "South Sudan"[tiab] OR sudan[tiab] OR sudan[mesh] OR swaziland[MeSH] OR swaziland[tiab] OR tanzania[MeSH] OR tanzania[tiab] OR togo[tiab] OR togo[mesh] OR uganda[MeSH] OR uganda[tiab] OR zambia[MeSH] OR zambia[tiab] OR zimbabwe[MeSH] OR zimbabwe[tiab] OR "Africa South of the Sahara"[Mesh] OR "sub Saharan Africa"[tiab] OR "subsaharan Africa"[tiab])

AND medline[sb]

AND English[lang] AND ("2010/01/01"[Date - Publication] : "2021/12/31"[Date - Publication])

NOT (letter[ptyp] OR editorial[ptyp] OR comment[ptyp] OR "Congress" [Publication Type] OR "Consensus Development Conference" [Publication Type] OR letter[tw] OR editorial[tw] OR commentary[tiab] OR “conference abstract*”[tiab] OR “conference proceeding*”[tiab] OR news[ptyp] OR “retracted publication”[ptyp] OR “retraction of publication”[ptyp] OR “retraction of publication”[tiab] OR “retraction notice”[ti] OR erratum[tw])

**With NARROW implementation science search strategy**

(Adolescent[mesh] OR adolescent[tiab] OR adolescents[tiab] OR adolescence[tiab] OR teenager[tiab] OR teenagers[tiab] OR teen[tiab] OR teens[tiab] OR youth[tiab] OR youths[tiab] OR “young adult”[tiab] OR “young adults”[tiab] OR “Young Adult”[mesh]) AND ((prevention[tiab] OR prevent*[tiab] OR preventative[tiab] OR screen*[tiab] OR screening*[tiab] OR treatment*[tiab] OR treat*[tiab] OR care[tiab] OR therapy[tiab] OR therapies[tiab] OR therapeutic*[tiab] OR "Preventive Health Services"[Mesh] OR "Primary Prevention"[Mesh] OR "Secondary Prevention"[Mesh] OR "Tertiary Prevention"[Mesh] OR "prevention and control"[Subheading] OR "Therapeutics"[Mesh] OR "therapy" [Subheading]) AND (HIV[tiab] OR hiv1[tiab] OR hiv2[tiab] OR hiv-1*[tiab] OR hiv-2*[tiab] OR HIV[mesh] OR AIDS[tiab] OR “human immunodeficiency virus”[tiab] OR “human immunodeficiency viruses”[tiab] OR “human immunedeficiency virus”[tiab] OR “human immuno-deficiency virus”[tiab] OR “acquired immunodeficiency syndrome”[tiab] OR “acquired immunodeficiency syndromes”[tiab] OR “acquired immunedeficiency syndrome”[tiab] OR “acquired immuno-deficiency syndrome”[tiab] OR “acquired immuno-deficiency syndromes”[tiab] OR “acquired immune-deficiency syndrome”[tiab] OR “acquired immune-deficiency syndromes”[tiab] OR “HIV Infections”[majr])) AND (implementing[ti] OR implementation[ti] OR “implementation theory”[tiab] OR “implementation theories”[tiab] OR "implementation science"[tiab] OR "implementation research"[tiab] OR “implementation intervention”[tiab] OR “implementation interventions”[tiab]OR "implementation strategy"[tiab] OR "implementation strategies"[tiab] OR “implementation framework”[tiab] OR “implementation frameworks”[tiab] OR “implementation barrier”[tiab] OR “implementation barriers”[tiab] OR “intervention research”[tiab] OR “intervention oriented research”[tiab] OR “improvement science”[tiab] OR "dissemination research"[tiab] OR "dissemination science"[tiab] OR "translational science"[tiab] OR "diffusion of innovation"[tiab] OR "impact evaluation"[tiab] OR “impact evaluations”[tiab] OR “impact research”[tiab] OR “knowledge transfer”[tiab] OR “transfer knowledge”[tiab] OR “knowledge exchange”[tiab] OR “knowledge translation”[tiab] OR “knowledge to action”[tiab] OR “research utilization”[tiab] OR (“organizational readiness”[tiab] AND change[tiab]) OR "ecological framework"[tiab] OR "ecological frameworks"[tiab] OR "Re-AIM"[tiab] OR "reach effectiveness adoption implementation maintenance"[tiab] OR PARIHS[tiab] OR "promoting action on research implementation in health services"[tiab] OR CFIR[tiab] OR "PRECEDE-PROCEED"[tiab] OR “interactive systems framework*”[tiab] OR “Theoretical Domains Framework*”[tiab] OR AIF[tiab] OR “Active Implementation Framework*”[tiab] OR “Consolidated Framework for Implementation Research”[tiab] OR NICS[tiab] OR PRISM[tiab] OR SURE[tiab] OR TDF[tiab] OR “Theoretical Domains Framework*”[tiab] OR TICD[tiab] OR “Exploration Preparation Implementation Sustainment framework”[tiab] OR EPIS[tiab] OR "Implementation Science"[Mesh] OR "Health Plan Implementation"[Mesh]) AND (angola[MeSH] OR angola[tiab] OR benin[tiab] OR benin[mesh] OR botswana[tiab] OR Botswana[mesh] OR "Burkina faso"[mesh] OR "burkina faso"[tiab] OR burundi[MeSH] OR burundi[tiab] OR "cabo verde"[tiab] OR cameroon[MeSH] OR cameroon[tiab] OR "central African republic"[tiab] OR "central African republic"[mesh] OR chad[tiab] OR chad[mesh] OR comoros[tiab] OR comoros[mesh] OR "Cote d'Ivoire"[mesh] OR "Cote d'Ivoire"[tiab] OR "Ivory Coast"[tiab] OR "Democratic Republic of the Congo"[tiab] OR congo[MeSH] OR congo[tiab] OR "republic of the congo"[tiab] OR "equatorial guinea"[tiab] OR "equatorial guinea"[mesh] OR Eritrea[mesh] OR Eritrea[tiab] OR Eswatini[tiab] OR Ethiopia[tiab] OR Ethiopia[mesh] OR gabon[tiab] OR gabon[mesh] OR gambia[mesh] OR gambia[tiab] OR ghana[MeSH] OR ghana[tiab] OR guinea[tiab] OR guinea[mesh] OR "guinea-bissau"[tiab] OR "guinea-bissau"[mesh] OR kenya[MeSH] OR kenya[tiab] OR lesotho[MeSH] OR lesotho[tiab] OR Liberia[tiab] OR Liberia[mesh] OR Madagascar[tiab] OR Madagascar[mesh] OR malawi[MeSH] OR malawi[tiab] OR mali[tiab] OR mali[mesh] OR Mauritania[mesh] OR Mauritania[tiab] OR Mauritius[mesh] OR Mauritius[tiab] OR mozambique[MeSH] OR mozambique[tiab] OR namibia[MeSH] OR namibia[tiab] OR niger[tiab] OR niger[mesh] OR nigeria[MeSH] OR nigeria[tiab] OR rwanda[MeSH] OR rwanda[tiab] OR "sao tome and principe"[tiab] OR "sao tome"[tiab] OR "sao tome and principe"[mesh] OR Senegal[tiab] OR Senegal[mesh] OR Seychelles[tiab] OR Seychelles[mesh] OR "sierra leone"[tiab] OR "sierra leone"[mesh] OR Somalia[tiab] OR Somalia[mesh] OR Somaliland[tiab] OR "South Africa"[tiab] OR "south Africa"[mesh] OR "south sudan"[mesh] OR "South Sudan"[tiab] OR sudan[tiab] OR sudan[mesh] OR swaziland[MeSH] OR swaziland[tiab] OR tanzania[MeSH] OR tanzania[tiab] OR togo[tiab] OR togo[mesh] OR uganda[MeSH] OR uganda[tiab] OR zambia[MeSH] OR zambia[tiab] OR zimbabwe[MeSH] OR zimbabwe[tiab] OR "Africa South of the Sahara"[Mesh] OR "sub Saharan Africa"[tiab] OR "subsaharan Africa"[tiab])

AND medline[sb]

AND English[lang] AND ("2010/01/01"[Date - Publication] : "2021/12/31"[Date - Publication])

NOT (letter[ptyp] OR editorial[ptyp] OR comment[ptyp] OR "Congress" [Publication Type] OR "Consensus Development Conference" [Publication Type] OR letter[tw] OR editorial[tw] OR commentary[tiab] OR “conference abstract*”[tiab] OR “conference proceeding*”[tiab] OR news[ptyp] OR “retracted publication”[ptyp] OR “retraction of publication”[ptyp] OR “retraction of publication”[tiab] OR “retraction notice”[ti] OR erratum[tw])

**Database:** Embase
**Vendor:** Elsevier

**Date of Search:** October 28, 2020 & September 15, 2021
**Limits:** Language: English; Publication date: 2010 – 2021; Source: Embase & Embase Classic

**Notes:** Use Advanced search. Limit to title and abstract fields, title field, and EMTREE fields for terms. Run main search first and then apply the limits for language, year, and source to those results. Use the search strategy to exclude publication types of no interest.

**With BROAD implementation science search strategy**

(adolescent:ti,ab OR adolescents:ti,ab OR adolescence:ti,ab OR teenager:ti,ab OR teenagers:ti,ab OR teen:ti,ab OR teens:ti,ab OR youth:ti,ab OR youths:ti,ab OR “young adult”:ti,ab OR “young adults”:ti,ab OR 'adolescent'/exp OR 'young adult'/exp) AND ((prevention:ti,ab OR prevent*:ti,ab OR preventative:ti,ab OR screen*:ti,ab OR screening*:ti,ab OR treatment*:ti,ab OR treat*:ti,ab OR care:ti,ab OR therapy:ti,ab OR therapies:ti,ab OR therapeutic*:ti,ab OR 'preventive health service'/exp OR 'primary prevention'/exp OR 'secondary prevention'/exp OR 'tertiary prevention'/exp OR 'therapy'/de OR 'prevention and control'/exp) AND (HIV:ti,ab OR hiv1:ti,ab OR hiv2:ti,ab OR hiv-1*:ti,ab OR hiv-2*:ti,ab OR AIDS:ti,ab OR “human immunodeficiency virus”:ti,ab OR “human immunodeficiency viruses”:ti,ab OR “human immunedeficiency virus”:ti,ab OR “human immunedeficiency viruses”:ti,ab OR “human immuno-deficiency virus”:ti,ab OR “human immuno-deficiency viruses”:ti,ab OR “acquired immunodeficiency syndrome”:ti,ab OR “acquired immunodeficiency syndromes”:ti,ab OR “acquired immunedeficiency syndrome”:ti,ab OR “acquired immunedeficiency syndromes”:ti,ab OR “acquired immuno-deficiency syndrome”:ti,ab OR “acquired immuno-deficiency syndromes”:ti,ab OR “acquired immune-deficiency syndrome”:ti,ab OR “acquired immune-deficiency syndromes”:ti,ab OR 'Human immunodeficiency virus'/exp OR 'Human immunodeficiency virus 1'/exp OR 'Human immunodeficiency virus 2'/exp OR 'acquired immune deficiency syndrome'/exp OR 'Human immunodeficiency virus infection'/exp OR 'acute HIV infection'/exp)) AND (implementing:ti OR implementation:ti OR “implementation theory”:ti,ab OR “implementation theories”:ti,ab OR "implementation science":ti,ab OR "implementation research":ti,ab OR “implementation intervention”:ti,ab OR “implementation interventions”:ti,ab OR "implementation strategy":ti,ab OR "implementation strategies":ti,ab OR “implementation framework”:ti,ab OR “implementation frameworks”:ti,ab OR “implementation barrier”:ti,ab OR “implementation barriers”:ti,ab OR “intervention research”:ti,ab OR “intervention oriented research”:ti,ab OR “improvement science”:ti,ab OR "dissemination research":ti,ab OR "dissemination science":ti,ab OR "translational research":ti,ab OR "diffusion of innovation":ti,ab OR "impact evaluation":ti,ab OR “impact evaluations”:ti,ab OR “impact research”:ti,ab OR “knowledge transfer”:ti,ab OR “transfer knowledge”:ti,ab OR “knowledge translation”:ti,ab OR “knowledge exchange”:ti,ab OR “knowledge to action”:ti,ab OR acceptability:ti,ab OR adoption:ti,ab OR appropriateness:ti,ab OR feasibility:ti,ab OR fidelity:ti,ab OR "implementation cost":ti,ab OR "implementation costs":ti,ab OR sustainability:ti,ab OR "implementation outcome":ti,ab OR "implementation outcomes":ti,ab OR "implementation process":ti,ab OR "implementation processes":ti,ab OR "effectiveness-implementation hybrid":ti,ab OR “community based implementation”:ti,ab OR "ecological framework":ti,ab OR "ecological frameworks":ti,ab OR (“organizational readiness”:ti,ab AND change:ti,ab) OR "Re-AIM":ti,ab OR "reach effectiveness adoption implementation maintenance":ti,ab OR PARIHS:ti,ab OR "promoting action on research implementation in health services":ti,ab OR CFIR:ti,ab OR "PRECEDE PROCEED":ti,ab OR “interactive systems framework”:ti,ab OR “interactive systems frameworks”:ti,ab OR “Theoretical Domains Framework”:ti,ab OR “Theoretical Domains Frameworks”:ti,ab OR AIF:ti,ab OR “Active Implementation Framework”:ti,ab OR “Active Implementation Frameworks”:ti,ab OR “Consolidated Framework for Implementation Research”:ti,ab OR NICS:ti,ab OR PRISM:ti,ab OR SURE:ti,ab OR TDF:ti,ab OR “Theoretical Domains Framework”:ti,ab OR “Theoretical Domains Frameworks”:ti,ab OR TICD:ti,ab OR “Exploration Preparation Implementation Sustainment framework”:ti,ab OR EPIS:ti,ab OR "Health Plan Implementation":ti,ab OR 'implementation science'/exp OR 'knowledge translation'/exp OR 'knowledge transfer'/exp OR 'health care planning'/exp) AND (angola:ti,ab OR benin:ti,ab OR botswana:ti,ab OR "burkina faso":ti,ab OR burundi:ti,ab OR "cabo verde":ti,ab OR cameroon:ti,ab OR "central African republic":ti,ab OR chad:ti,ab OR comoros:ti,ab OR "Cote d Ivoire":ti,ab OR "Ivory Coast":ti,ab OR "Democratic Republic of the Congo":ti,ab OR congo:ti,ab OR "republic of the congo":ti,ab OR "equatorial guinea":ti,ab OR Eritrea:ti,ab OR Eswatini:ti,ab OR Ethiopia:ti,ab OR gabon:ti,ab OR gambia:ti,ab OR ghana:ti,ab OR guinea:ti,ab OR "guinea-bissau":ti,ab OR kenya:ti,ab OR lesotho:ti,ab OR Liberia:ti,ab OR Madagascar:ti,ab OR malawi:ti,ab OR mali:ti,ab OR Mauritania:ti,ab OR Mauritius:ti,ab OR mozambique:ti,ab OR namibia:ti,ab OR niger:ti,ab OR nigeria:ti,ab OR rwanda:ti,ab OR "sao tome and principe":ti,ab OR "sao tome":ti,ab OR Senegal:ti,ab OR Seychelles:ti,ab OR "sierra leone":ti,ab OR Somalia:ti,ab OR Somaliland:ti,ab OR "South Africa":ti,ab OR "South Sudan":ti,ab OR sudan:ti,ab OR swaziland:ti,ab OR tanzania:ti,ab OR togo:ti,ab OR uganda:ti,ab OR zambia:ti,ab OR zimbabwe:ti,ab OR "sub Saharan Africa":ti,ab OR "subsaharan Africa":ti,ab OR 'Africa south of the Sahara'/exp OR 'Angola'/exp OR 'Benin'/exp OR 'Botswana'/exp OR 'Burkina Faso'/exp OR 'Burundi'/exp OR 'Cameroon'/exp OR 'Cape Verde'/exp OR 'Central Africa'/exp OR 'Central African Republic'/exp OR 'Chad'/exp OR 'Comoros'/exp OR 'Congo'/exp OR 'Cote d`Ivoire'/exp OR 'Democratic Republic Congo'/exp OR 'Equatorial Guinea'/exp OR 'Eritrea'/exp OR 'Eswatini'/exp OR 'Ethiopia'/exp OR 'Gabon'/exp OR 'Gambia'/exp OR 'Ghana'/exp OR 'Guinea'/exp OR 'Guinea-Bissau'/exp OR 'Kenya'/exp OR 'Lesotho'/exp OR 'Liberia'/exp OR 'Madagascar'/exp OR 'Malawi'/exp OR 'Mali'/exp OR 'Mozambique'/exp OR 'Namibia'/exp OR 'Niger'/exp OR 'Nigeria'/exp OR 'Rwanda'/exp OR 'Sahel'/exp OR 'Senegal'/exp OR 'Sierra Leone'/exp OR 'Somalia'/exp OR 'South Africa'/exp OR 'South Sudan'/exp OR 'Sudan'/exp OR 'Tanzania'/exp OR 'Togo'/exp OR 'Uganda'/exp OR 'Zambia'/exp OR 'Zimbabwe'/exp)

AND [english]/lim AND ([embase]/lim OR [embase classic]/lim) AND [2010-2021]/py

NOT ([conference abstract]/lim OR [conference paper]/lim OR [conference review]/lim OR [editorial]/lim OR [erratum]/lim OR [letter]/lim OR [note]/lim OR [short survey]/lim OR 'retraction notice'/exp OR 'erratum'/exp OR 'letter'/exp OR 'conference paper'/exp OR 'editorial'/exp OR 'note'/exp OR 'short survey'/exp OR 'conference abstract'/exp OR editorial:ti,ab OR commentary:ti,ab OR “conference abstract*”:ti,ab OR “conference proceeding*”:ti,ab OR “retracted publication”:ti,ab OR “retraction of publication”:ti,ab OR “retraction of publication”:ab,ti OR “retraction notice”:ti OR erratum:ti,ab)

**With NARROW implementation science search strategy**

(adolescent:ti,ab OR adolescents:ti,ab OR adolescence:ti,ab OR teenager:ti,ab OR teenagers:ti,ab OR teen:ti,ab OR teens:ti,ab OR youth:ti,ab OR youths:ti,ab OR “young adult”:ti,ab OR “young adults”:ti,ab OR 'adolescent'/exp OR 'young adult'/exp) AND ((prevention:ti,ab OR prevent*:ti,ab OR preventative:ti,ab OR screen*:ti,ab OR screening*:ti,ab OR treatment*:ti,ab OR treat*:ti,ab OR care:ti,ab OR therapy:ti,ab OR therapies:ti,ab OR therapeutic*:ti,ab OR 'preventive health service'/exp OR 'primary prevention'/exp OR 'secondary prevention'/exp OR 'tertiary prevention'/exp OR 'therapy'/de OR 'prevention and control'/exp) AND (HIV:ti,ab OR hiv1:ti,ab OR hiv2:ti,ab OR hiv-1*:ti,ab OR hiv-2*:ti,ab OR AIDS:ti,ab OR “human immunodeficiency virus”:ti,ab OR “human immunodeficiency viruses”:ti,ab OR “human immunedeficiency virus”:ti,ab OR “human immunedeficiency viruses”:ti,ab OR “human immuno-deficiency virus”:ti,ab OR “human immuno-deficiency viruses”:ti,ab OR “acquired immunodeficiency syndrome”:ti,ab OR “acquired immunodeficiency syndromes”:ti,ab OR “acquired immunedeficiency syndrome”:ti,ab OR “acquired immunedeficiency syndromes”:ti,ab OR “acquired immuno-deficiency syndrome”:ti,ab OR “acquired immuno-deficiency syndromes”:ti,ab OR “acquired immune-deficiency syndrome”:ti,ab OR “acquired immune-deficiency syndromes”:ti,ab OR 'Human immunodeficiency virus'/exp OR 'Human immunodeficiency virus 1'/exp OR 'Human immunodeficiency virus 2'/exp OR 'acquired immune deficiency syndrome'/exp OR 'Human immunodeficiency virus infection'/exp OR 'acute HIV infection'/exp)) AND (implementing:ti OR implementation:ti OR “implementation theory”:ti,ab OR “implementation theories”:ti,ab OR "implementation science":ti,ab OR "implementation research":ti,ab OR “implementation intervention”:ti,ab OR “implementation interventions”:ti,ab OR "implementation strategy":ti,ab OR "implementation strategies":ti,ab OR “implementation framework”:ti,ab OR “implementation frameworks”:ti,ab OR “implementation barrier”:ti,ab OR “implementation barriers”:ti,ab OR “intervention research”:ti,ab OR “intervention oriented research”:ti,ab OR “improvement science”:ti,ab OR "dissemination research":ti,ab OR "dissemination science":ti,ab OR "translational science":ti,ab OR "diffusion of innovation":ti,ab OR "impact evaluation":ti,ab OR “impact evaluations”:ti,ab OR “impact research”:ti,ab OR “knowledge transfer”:ti,ab OR “transfer knowledge”:ti,ab OR “knowledge exchange”:ti,ab OR “knowledge translation”:ti,ab OR “knowledge to action”:ti,ab OR “research utilization”:ti,ab OR (“organizational readiness”:ti,ab AND change:ti,ab) OR "ecological framework":ti,ab OR "ecological frameworks":ti,ab OR "Re-AIM":ti,ab OR "reach effectiveness adoption implementation maintenance":ti,ab OR PARIHS:ti,ab OR "promoting action on research implementation in health services":ti,ab OR CFIR:ti,ab OR "PRECEDE PROCEED":ti,ab OR “interactive systems framework”:ti,ab OR “Theoretical Domains Framework”:ti,ab OR “interactive systems frameworks”:ti,ab OR “Theoretical Domains Frameworks”:ti,ab OR AIF:ti,ab OR “Active Implementation Framework”:ti,ab OR “Active Implementation Frameworks”:ti,ab OR “Consolidated Framework for Implementation Research”:ti,ab OR NICS:ti,ab OR PRISM:ti,ab OR SURE:ti,ab OR TDF:ti,ab OR “Theoretical Domains Framework”:ti,ab OR “Theoretical Domains Frameworks”:ti,ab OR TICD:ti,ab OR “Exploration Preparation Implementation Sustainment framework”:ti,ab OR EPIS:ti,ab OR "Health Plan Implementation":ti,ab OR 'implementation science'/exp OR 'knowledge translation'/exp OR 'knowledge transfer'/exp OR 'health care planning'/exp) AND (angola:ti,ab OR benin:ti,ab OR botswana:ti,ab OR "burkina faso":ti,ab OR burundi:ti,ab OR "cabo verde":ti,ab OR cameroon:ti,ab OR "central African republic":ti,ab OR chad:ti,ab OR comoros:ti,ab OR "Cote d Ivoire":ti,ab OR "Ivory Coast":ti,ab OR "Democratic Republic of the Congo":ti,ab OR congo:ti,ab OR "republic of the congo":ti,ab OR "equatorial guinea":ti,ab OR Eritrea:ti,ab OR Eswatini:ti,ab OR Ethiopia:ti,ab OR gabon:ti,ab OR gambia:ti,ab OR ghana:ti,ab OR guinea:ti,ab OR "guinea-bissau":ti,ab OR kenya:ti,ab OR lesotho:ti,ab OR Liberia:ti,ab OR Madagascar:ti,ab OR malawi:ti,ab OR mali:ti,ab OR Mauritania:ti,ab OR Mauritius:ti,ab OR mozambique:ti,ab OR namibia:ti,ab OR niger:ti,ab OR nigeria:ti,ab OR rwanda:ti,ab OR "sao tome and principe":ti,ab OR "sao tome":ti,ab OR Senegal:ti,ab OR Seychelles:ti,ab OR "sierra leone":ti,ab OR Somalia:ti,ab OR Somaliland:ti,ab OR "South Africa":ti,ab OR "South Sudan":ti,ab OR sudan:ti,ab OR swaziland:ti,ab OR tanzania:ti,ab OR togo:ti,ab OR uganda:ti,ab OR zambia:ti,ab OR zimbabwe:ti,ab OR "sub Saharan Africa":ti,ab OR "subsaharan Africa":ti,ab OR 'Africa south of the Sahara'/exp OR 'Angola'/exp OR 'Benin'/exp OR 'Botswana'/exp OR 'Burkina Faso'/exp OR 'Burundi'/exp OR 'Cameroon'/exp OR 'Cape Verde'/exp OR 'Central Africa'/exp OR 'Central African Republic'/exp OR 'Chad'/exp OR 'Comoros'/exp OR 'Congo'/exp OR 'Cote d`Ivoire'/exp OR 'Democratic Republic Congo'/exp OR 'Equatorial Guinea'/exp OR 'Eritrea'/exp OR 'Eswatini'/exp OR 'Ethiopia'/exp OR 'Gabon'/exp OR 'Gambia'/exp OR 'Ghana'/exp OR 'Guinea'/exp OR 'Guinea-Bissau'/exp OR 'Kenya'/exp OR 'Lesotho'/exp OR 'Liberia'/exp OR 'Madagascar'/exp OR 'Malawi'/exp OR 'Mali'/exp OR 'Mozambique'/exp OR 'Namibia'/exp OR 'Niger'/exp OR 'Nigeria'/exp OR 'Rwanda'/exp OR 'Sahel'/exp OR 'Senegal'/exp OR 'Sierra Leone'/exp OR 'Somalia'/exp OR 'South Africa'/exp OR 'South Sudan'/exp OR 'Sudan'/exp OR 'Tanzania'/exp OR 'Togo'/exp OR 'Uganda'/exp OR 'Zambia'/exp OR 'Zimbabwe'/exp)

AND [english]/lim AND ([embase]/lim OR [embase classic]/lim) AND [2010-2021]/py

NOT ([conference abstract]/lim OR [conference paper]/lim OR [conference review]/lim OR [editorial]/lim OR [erratum]/lim OR [letter]/lim OR [note]/lim OR [short survey]/lim OR 'retraction notice'/exp OR 'erratum'/exp OR 'letter'/exp OR 'conference paper'/exp OR 'editorial'/exp OR 'note'/exp OR 'short survey'/exp OR 'conference abstract'/exp OR editorial:ti,ab OR commentary:ti,ab OR “conference abstract*”:ti,ab OR “conference proceeding*”:ti,ab OR “retracted publication”:ti,ab OR “retraction of publication”:ti,ab OR “retraction of publication”:ab,ti OR “retraction notice”:ti OR erratum:ti,ab)

**Database:** Web of Science: Core Collection
**Vendor:** Clarivate Analytics

**Date of Search:** October 28, 2020 & September 15, 2021
**Limits:** Language: English; Publication date: 2010 – 2021

**Notes:** Use Advanced Search. Limit terms to the Topic field. Run the main search first and apply the language and publication year filters. The to the results use the filters on the left of the results screen to exclude specific Document Types not of interest (proceedings paper, meeting abstract, editorial material) using the filter and then select exclude.

**With BROAD implementation science search strategy**

TS=((adolescent OR adolescents OR adolescence OR teenager OR teenagers OR teen OR teens OR youth OR youths OR “young adult” OR “young adults”) AND ((prevention OR prevent* OR preventative OR screen* OR screening* OR treatment* OR treat* OR care OR therapy OR therapies OR therapeutic* OR “preventive health service” OR “primary prevention” OR “secondary prevention” OR “tertiary prevention” OR “prevention and control”) AND (HIV OR hiv1 OR hiv2 OR hiv-1* OR hiv-2* OR AIDS OR “human immunodeficiency virus” OR “human immunodeficiency viruses” OR “human immunedeficiency virus” OR “human immunedeficiency viruses” OR “human immuno-deficiency virus” OR “human immuno-deficiency viruses” OR “acquired immunodeficiency syndrome” OR “acquired immunodeficiency syndromes” OR “acquired immunedeficiency syndrome” OR “acquired immunedeficiency syndromes” OR “acquired immuno-deficiency syndrome” OR “acquired immuno-deficiency syndromes” OR “acquired immune-deficiency syndrome” OR “acquired immune-deficiency syndromes” OR “acquired immune deficiency syndrome”)) AND (implementing OR implementation OR “implementation theory” OR “implementation theories” OR "implementation science" OR "implementation research" OR “implementation intervention” OR “implementation interventions” OR "implementation strategy" OR "implementation strategies" OR “implementation framework” OR “implementation frameworks” OR “implementation barrier” OR “implementation barriers” OR “intervention research” OR “intervention oriented research” OR “improvement science” OR "dissemination research" OR "dissemination science" OR "translational research" OR "diffusion of innovation" OR "impact evaluation" OR “impact evaluations” OR “impact research” OR “knowledge transfer” OR “transfer knowledge” OR “knowledge translation” OR “knowledge exchange” OR “knowledge to action” OR acceptability OR adoption OR appropriateness OR feasibility OR fidelity OR "implementation cost" OR "implementation costs" OR sustainability OR "implementation outcome" OR "implementation outcomes" OR "implementation process" OR "implementation processes" OR "effectiveness-implementation hybrid" OR “community based implementation” OR "ecological framework" OR "ecological frameworks" OR (“organizational readiness” AND change) OR "Re-AIM" OR "reach effectiveness adoption implementation maintenance" OR PARIHS OR "promoting action on research implementation in health services" OR CFIR OR "PRECEDE PROCEED" OR “interactive systems framework” OR “interactive systems frameworks” OR “Theoretical Domains Framework” OR “Theoretical Domains Frameworks” OR AIF OR “Active Implementation Framework” OR “Active Implementation Frameworks” OR “Consolidated Framework for Implementation Research” OR NICS OR PRISM OR SURE OR TDF OR “Theoretical Domains Framework” OR “Theoretical Domains Frameworks” OR TICD OR “Exploration Preparation Implementation Sustainment framework” OR EPIS OR "Health Plan Implementation") AND (angola OR benin OR botswana OR "burkina faso" OR burundi OR "cabo verde" OR cameroon OR "central African republic" OR chad OR comoros OR "Cote d Ivoire" OR "Ivory Coast" OR "Democratic Republic of the Congo" OR congo OR "republic of the congo" OR "equatorial guinea" OR Eritrea OR Eswatini OR Ethiopia OR gabon OR gambia OR ghana OR guinea OR "guinea-bissau" OR kenya OR lesotho OR Liberia OR Madagascar OR malawi OR mali OR Mauritania OR Mauritius OR mozambique OR namibia OR niger OR nigeria OR rwanda OR "sao tome and principe" OR "sao tome" OR Senegal OR Seychelles OR "sierra leone" OR Somalia OR Somaliland OR "South Africa" OR "South Sudan" OR sudan OR swaziland OR tanzania OR togo OR uganda OR zambia OR zimbabwe OR "sub Saharan Africa" OR "subsaharan Africa"))

**With NARROW implementation science search strategy**

TS=((adolescent OR adolescents OR adolescence OR teenager OR teenagers OR teen OR teens OR youth OR youths OR “young adult” OR “young adults”) AND ((prevention OR prevent* OR preventative OR screen* OR screening* OR treatment* OR treat* OR care OR therapy OR therapies OR therapeutic* OR “preventive health service” OR “primary prevention” OR “secondary prevention” OR “tertiary prevention” OR “prevention and control”) AND (HIV OR hiv1 OR hiv2 OR hiv-1* OR hiv-2* OR AIDS OR “human immunodeficiency virus” OR “human immunodeficiency viruses” OR “human immunedeficiency virus” OR “human immunedeficiency viruses” OR “human immuno-deficiency virus” OR “human immuno-deficiency viruses” OR “acquired immunodeficiency syndrome” OR “acquired immunodeficiency syndromes” OR “acquired immunedeficiency syndrome” OR “acquired immunedeficiency syndromes” OR “acquired immuno-deficiency syndrome” OR “acquired immuno-deficiency syndromes” OR “acquired immune-deficiency syndrome” OR “acquired immune-deficiency syndromes” OR “acquired immune deficiency syndrome”)) AND (implementing OR implementation OR “implementation theory” OR “implementation theories” OR "implementation science" OR "implementation research" OR “implementation intervention” OR “implementation interventions” OR "implementation strategy" OR "implementation strategies" OR “implementation framework” OR “implementation frameworks” OR “implementation barrier” OR “implementation barriers” OR “intervention research” OR “intervention oriented research” OR “improvement science” OR "dissemination research" OR "dissemination science" OR "translational science" OR "diffusion of innovation" OR "impact evaluation" OR “impact evaluations” OR “impact research” OR “knowledge transfer” OR “transfer knowledge” OR “knowledge exchange” OR “knowledge translation” OR “knowledge to action” OR “research utilization” OR (“organizational readiness” AND change) OR "ecological framework" OR "ecological frameworks" OR "Re-AIM" OR "reach effectiveness adoption implementation maintenance" OR PARIHS OR "promoting action on research implementation in health services" OR CFIR OR "PRECEDE PROCEED" OR “interactive systems framework” OR “Theoretical Domains Framework” OR “interactive systems frameworks” OR “Theoretical Domains Frameworks” OR AIF OR “Active Implementation Framework” OR “Active Implementation Frameworks” OR “Consolidated Framework for Implementation Research” OR NICS OR PRISM OR SURE OR TDF OR “Theoretical Domains Framework” OR “Theoretical Domains Frameworks” OR TICD OR “Exploration Preparation Implementation Sustainment framework” OR EPIS OR "Health Plan Implementation”) AND (angola OR benin OR botswana OR "burkina faso" OR burundi OR "cabo verde" OR cameroon OR "central African republic" OR chad OR comoros OR "Cote d Ivoire" OR "Ivory Coast" OR "Democratic Republic of the Congo" OR congo OR "republic of the congo" OR "equatorial guinea" OR Eritrea OR Eswatini OR Ethiopia OR gabon OR gambia OR ghana OR guinea OR "guinea-bissau" OR kenya OR lesotho OR Liberia OR Madagascar OR malawi OR mali OR Mauritania OR Mauritius OR mozambique OR namibia OR niger OR nigeria OR rwanda OR "sao tome and principe" OR "sao tome" OR Senegal OR Seychelles OR "sierra leone" OR Somalia OR Somaliland OR "South Africa" OR "South Sudan" OR sudan OR swaziland OR tanzania OR togo OR uganda OR zambia OR zimbabwe OR "sub Saharan Africa" OR "subsaharan Africa"))

**Database:** Scopus
**Vendor:** Elsevier

**Date of Search:** October 28, 2020 & September 15, 2021
**Limits:** Language: English; Publication date: 2010 – 2021

**Notes:** Use Advanced search. Limit to the title, abstract, and keyword fields for terms. Run main search first and then exclude the specific Document Types (editorials, conference paper, note, letter, book chapter) using the filter and then select exclude. Then to these results apply the limits for language and year to those results.

**With BROAD implementation science search strategy**

Title-Abs-Key((adolescent OR adolescents OR adolescence OR teenager OR teenagers OR teen OR teens OR youth OR youths OR {young adult} OR {young adults}) AND ((prevention OR prevent* OR preventative OR screen* OR screening* OR treatment* OR treat* OR care OR therapy OR therapies OR therapeutic* OR {preventive health service} OR {primary prevention} OR {secondary prevention} OR {tertiary prevention} OR {prevention and control}) AND (HIV OR hiv1 OR hiv2 OR hiv-1* OR hiv-2* OR AIDS OR {human immunodeficiency virus} OR {human immunodeficiency viruses} OR {human immunedeficiency virus} OR {human immunedeficiency viruses} OR {human immuno-deficiency virus} OR {human immuno-deficiency viruses} OR {acquired immunodeficiency syndrome} OR {acquired immunodeficiency syndromes} OR {acquired immunedeficiency syndrome} OR {acquired immunedeficiency syndromes} OR {acquired immuno-deficiency syndrome} OR {acquired immuno-deficiency syndromes} OR {acquired immune-deficiency syndrome} OR {acquired immune-deficiency syndromes} OR {acquired immune deficiency syndrome})) AND (implementing OR implementation OR {implementation theory} OR {implementation theories} OR {implementation science} OR {implementation research} OR {implementation intervention} OR {implementation interventions} OR {implementation strategy} OR {implementation strategies} OR {implementation framework} OR {implementation frameworks} OR {implementation barrier} OR {implementation barriers} OR {intervention research} OR {intervention oriented research} OR {improvement science} OR {dissemination research} OR {dissemination science} OR {translational research} OR {diffusion of innovation} OR {impact evaluation} OR {impact evaluations} OR {impact research} OR {knowledge transfer} OR {transfer knowledge} OR {knowledge translation} OR {knowledge exchange} OR {knowledge to action} OR acceptability OR adoption OR appropriateness OR feasibility OR fidelity OR {implementation cost} OR {implementation costs} OR sustainability OR {implementation outcome} OR {implementation outcomes} OR {implementation process} OR {implementation processes} OR {effectiveness-implementation hybrid} OR {community based implementation} OR {ecological framework} OR {ecological frameworks} OR ({organizational readiness} AND change) OR {Re-AIM} OR {reach effectiveness adoption implementation maintenance} OR PARIHS OR {promoting action on research implementation in health services} OR CFIR OR {PRECEDE PROCEED} OR {interactive systems framework} OR {interactive systems frameworks} OR {Theoretical Domains Framework} OR {Theoretical Domains Frameworks} OR AIF OR {Active Implementation Framework} OR {Active Implementation Frameworks} OR {Consolidated Framework for Implementation Research} OR NICS OR PRISM OR SURE OR TDF OR {Theoretical Domains Framework} OR {Theoretical Domains Frameworks} OR TICD OR {Exploration Preparation Implementation Sustainment framework} OR EPIS OR {Health Plan Implementation}) AND (angola OR benin OR botswana OR {burkina faso} OR burundi OR {cabo verde} OR cameroon OR {central African republic} OR chad OR comoros OR {Cote d Ivoire} OR {Ivory Coast} OR {Democratic Republic of the Congo} OR congo OR {republic of the congo} OR {equatorial guinea} OR Eritrea OR Eswatini OR Ethiopia OR gabon OR gambia OR ghana OR guinea OR {guinea-bissau} OR kenya OR lesotho OR Liberia OR Madagascar OR malawi OR mali OR Mauritania OR Mauritius OR mozambique OR namibia OR niger OR nigeria OR rwanda OR {sao tome and principe} OR {sao tome} OR Senegal OR Seychelles OR {sierra leone} OR Somalia OR Somaliland OR {South Africa} OR {South Sudan} OR sudan OR swaziland OR tanzania OR togo OR uganda OR zambia OR zimbabwe OR {sub Saharan Africa} OR {subsaharan Africa}))

**With NARROW implementation science search strategy**

Title-Abs-Key((adolescent OR adolescents OR adolescence OR teenager OR teenagers OR teen OR teens OR youth OR youths OR {young adult} OR {young adults}) AND ((prevention OR prevent* OR preventative OR screen* OR screening* OR treatment* OR treat* OR care OR therapy OR therapies OR therapeutic* OR {preventive health service} OR {primary prevention} OR {secondary prevention} OR {tertiary prevention} OR {prevention and control}) AND (HIV OR hiv1 OR hiv2 OR hiv-1* OR hiv-2* OR AIDS OR {human immunodeficiency virus} OR {human immunodeficiency viruses} OR {human immunedeficiency virus} OR {human immunedeficiency viruses} OR {human immuno-deficiency virus} OR {human immuno-deficiency viruses} OR {acquired immunodeficiency syndrome} OR {acquired immunodeficiency syndromes} OR {acquired immunedeficiency syndrome} OR {acquired immunedeficiency syndromes} OR {acquired immuno-deficiency syndrome} OR {acquired immuno-deficiency syndromes} OR {acquired immune-deficiency syndrome} OR {acquired immune-deficiency syndromes} OR {acquired immune deficiency syndrome})) AND (implementing OR implementation OR {implementation theory} OR {implementation theories} OR {implementation science} OR {implementation research} OR {implementation intervention} OR {implementation interventions} OR {implementation strategy} OR {implementation strategies} OR {implementation framework} OR {implementation frameworks} OR {implementation barrier} OR {implementation barriers} OR {intervention research} OR {intervention oriented research} OR {improvement science} OR {dissemination research} OR {dissemination science} OR {translational science} OR {diffusion of innovation} OR {impact evaluation} OR {impact evaluations} OR {impact research} OR {knowledge transfer} OR {transfer knowledge} OR {knowledge exchange} OR {knowledge translation} OR {knowledge to action} OR {research utilization} OR ({organizational readiness} AND change) OR {ecological framework} OR {ecological frameworks} OR {Re-AIM} OR {reach effectiveness adoption implementation maintenance} OR PARIHS OR {promoting action on research implementation in health services} OR CFIR OR {PRECEDE PROCEED} OR {interactive systems framework} OR {Theoretical Domains Framework} OR {interactive systems frameworks} OR {Theoretical Domains Frameworks} OR AIF OR {Active Implementation Framework} OR {Active Implementation Frameworks} OR {Consolidated Framework for Implementation Research} OR NICS OR PRISM OR SURE OR TDF OR {Theoretical Domains Framework} OR {Theoretical Domains Frameworks} OR TICD OR {Exploration Preparation Implementation Sustainment framework} OR EPIS OR {Health Plan Implementation}) AND (angola OR benin OR botswana OR {burkina faso} OR burundi OR {cabo verde} OR cameroon OR {central African republic} OR chad OR comoros OR {Cote d Ivoire} OR {Ivory Coast} OR {Democratic Republic of the Congo} OR congo OR {republic of the congo} OR {equatorial guinea} OR Eritrea OR Eswatini OR Ethiopia OR gabon OR gambia OR ghana OR guinea OR {guinea-bissau} OR kenya OR lesotho OR Liberia OR Madagascar OR malawi OR mali OR Mauritania OR Mauritius OR mozambique OR namibia OR niger OR nigeria OR rwanda OR {sao tome and principe} OR {sao tome} OR Senegal OR Seychelles OR {sierra leone} OR Somalia OR Somaliland OR {South Africa} OR {South Sudan} OR sudan OR swaziland OR tanzania OR togo OR uganda OR zambia OR zimbabwe OR {sub Saharan Africa} OR {subsaharan Africa}))

**Database:** Global Health
**Vendor:** CAB Direct

**Date of Search:** October 28, 2020 & September 15, 2021
**Limits:** Language: English; Publication date: 2010 – 2020

**Notes:** Use Advanced search. Limit to the title, abstract, and CAB thesaurus fields for terms. Run main search components first and then combine each search component together. Then apply the limits for language and year to those results. Use the document type filter to limit to journal articles only.

**With BROAD implementation science search strategy**

#1 Article Title: (adolescent OR adolescents OR adolescence OR teenager OR teenagers OR teen OR teens OR youth OR youths OR “young adult” OR “young adults”)

#2 Abstract: (adolescent OR adolescents OR adolescence OR teenager OR teenagers OR teen OR teens OR youth OR youths OR “young adult” OR “young adults”)

#3 Subject term/Thesaurus: "adolescents" OR "young adults"

#4 #1 OR #2 OR #3

#5 Title: ((prevention OR prevent* OR preventative OR screen* OR screening* OR treatment* OR treat* OR care OR therapy OR therapies OR therapeutic* OR “preventive health service” OR “primary prevention” OR “secondary prevention” OR “tertiary prevention” OR “prevention and control”) AND (HIV OR hiv1 OR hiv2 OR hiv-1* OR hiv-2* OR AIDS OR “human immunodeficiency virus” OR “human immunodeficiency viruses” OR “human immunedeficiency virus” OR “human immunedeficiency viruses” OR “human immuno-deficiency virus” OR “human immuno-deficiency viruses” OR “acquired immunodeficiency syndrome” OR “acquired immunodeficiency syndromes” OR “acquired immunedeficiency syndrome” OR “acquired immunedeficiency syndromes” OR “acquired immuno-deficiency syndrome” OR “acquired immuno-deficiency syndromes” OR “acquired immune-deficiency syndrome” OR “acquired immune-deficiency syndromes” OR “acquired immune deficiency syndrome”))

#6 Abstract: ((prevention OR prevent* OR preventative OR screen* OR screening* OR treatment* OR treat* OR care OR therapy OR therapies OR therapeutic* OR “preventive health service” OR “primary prevention” OR “secondary prevention” OR “tertiary prevention” OR “prevention and control”) AND (HIV OR hiv1 OR hiv2 OR hiv-1* OR hiv-2* OR AIDS OR “human immunodeficiency virus” OR “human immunodeficiency viruses” OR “human immunedeficiency virus” OR “human immunedeficiency viruses” OR “human immuno-deficiency virus” OR “human immuno-deficiency viruses” OR “acquired immunodeficiency syndrome” OR “acquired immunodeficiency syndromes” OR “acquired immunedeficiency syndrome” OR “acquired immunedeficiency syndromes” OR “acquired immuno-deficiency syndrome” OR “acquired immuno-deficiency syndromes” OR “acquired immune-deficiency syndrome” OR “acquired immune-deficiency syndromes” OR “acquired immune deficiency syndrome”))

#7 Thesaurus: (("HIV" OR "human immunodeficiency viruses" OR "HIV-1" OR "HIV-1 infections" OR "HIV-2" OR "HIV-2 infections" OR "HIV-I" OR "HIV-II" OR "HIV infections" OR "AIDS" OR "acquired immune deficiency syndrome" OR "AIDS HTLV" OR "AIDS HTLV-I" OR "AIDS HTLV-II") AND ("prevention" OR "screening" OR "therapeutics" OR "therapy" OR "treatment"))

#8 #5 OR #6 OR #7

#9 Title: (implementing OR implementation OR “implementation theory” OR “implementation theories” OR "implementation science" OR "implementation research" OR “implementation intervention” OR “implementation interventions” OR "implementation strategy" OR "implementation strategies" OR “implementation framework” OR “implementation frameworks” OR “implementation barrier” OR “implementation barriers” OR “intervention research” OR “intervention oriented research” OR “improvement science” OR "dissemination research" OR "dissemination science" OR "translational research" OR "diffusion of innovation" OR "impact evaluation" OR “impact evaluations” OR “impact research” OR “knowledge transfer” OR “transfer knowledge” OR “knowledge translation” OR “knowledge exchange” OR “knowledge to action” OR acceptability OR adoption OR appropriateness OR feasibility OR fidelity OR "implementation cost" OR "implementation costs" OR sustainability OR "implementation outcome" OR "implementation outcomes" OR "implementation process" OR "implementation processes" OR "effectiveness-implementation hybrid" OR “community based implementation” OR "ecological framework" OR "ecological frameworks" OR (“organizational readiness” AND change) OR "Re-AIM" OR "reach effectiveness adoption implementation maintenance" OR PARIHS OR "promoting action on research implementation in health services" OR CFIR OR "PRECEDE PROCEED" OR “interactive systems framework” OR “interactive systems frameworks” OR “Theoretical Domains Framework” OR “Theoretical Domains Frameworks” OR AIF OR “Active Implementation Framework” OR “Active Implementation Frameworks” OR “Consolidated Framework for Implementation Research” OR NICS OR PRISM OR SURE OR TDF OR “Theoretical Domains Framework” OR “Theoretical Domains Frameworks” OR TICD OR “Exploration Preparation Implementation Sustainment framework” OR EPIS OR "Health Plan Implementation")

#10 Abstract: (implementing OR implementation OR “implementation theory” OR “implementation theories” OR "implementation science" OR "implementation research" OR “implementation intervention” OR “implementation interventions” OR "implementation strategy" OR "implementation strategies" OR “implementation framework” OR “implementation frameworks” OR “implementation barrier” OR “implementation barriers” OR “intervention research” OR “intervention oriented research” OR “improvement science” OR "dissemination research" OR "dissemination science" OR "translational research" OR "diffusion of innovation" OR "impact evaluation" OR “impact evaluations” OR “impact research” OR “knowledge transfer” OR “transfer knowledge” OR “knowledge translation” OR “knowledge exchange” OR “knowledge to action” OR acceptability OR adoption OR appropriateness OR feasibility OR fidelity OR "implementation cost" OR "implementation costs" OR sustainability OR "implementation outcome" OR "implementation outcomes" OR "implementation process" OR "implementation processes" OR "effectiveness-implementation hybrid" OR “community based implementation” OR "ecological framework" OR "ecological frameworks" OR (“organizational readiness” AND change) OR "Re-AIM" OR "reach effectiveness adoption implementation maintenance" OR PARIHS OR "promoting action on research implementation in health services" OR CFIR OR "PRECEDE PROCEED" OR “interactive systems framework” OR “interactive systems frameworks” OR “Theoretical Domains Framework” OR “Theoretical Domains Frameworks” OR AIF OR “Active Implementation Framework” OR “Active Implementation Frameworks” OR “Consolidated Framework for Implementation Research” OR NICS OR PRISM OR SURE OR TDF OR “Theoretical Domains Framework” OR “Theoretical Domains Frameworks” OR TICD OR “Exploration Preparation Implementation Sustainment framework” OR EPIS OR "Health Plan Implementation")

#11 Thesaurus: ("implementation" OR "implementation of research" OR "diffusion of research" OR "project implementation")

#12 #10 OR #11 OR #12

#13 Title: (angola OR benin OR botswana OR "burkina faso" OR burundi OR "cabo verde" OR cameroon OR "central African republic" OR chad OR comoros OR "Cote d Ivoire" OR "Ivory Coast" OR "Democratic Republic of the Congo" OR congo OR "republic of the congo" OR "equatorial guinea" OR Eritrea OR Eswatini OR Ethiopia OR gabon OR gambia OR ghana OR guinea OR "guinea-bissau" OR kenya OR lesotho OR Liberia OR Madagascar OR malawi OR mali OR Mauritania OR Mauritius OR mozambique OR namibia OR niger OR nigeria OR rwanda OR "sao tome and principe" OR "sao tome" OR Senegal OR Seychelles OR "sierra leone" OR Somalia OR Somaliland OR "South Africa" OR "South Sudan" OR sudan OR swaziland OR tanzania OR togo OR uganda OR zambia OR zimbabwe OR "sub Saharan Africa" OR "subsaharan Africa")

#14 Abstract: (angola OR benin OR botswana OR "burkina faso" OR burundi OR "cabo verde" OR cameroon OR "central African republic" OR chad OR comoros OR "Cote d Ivoire" OR "Ivory Coast" OR "Democratic Republic of the Congo" OR congo OR "republic of the congo" OR "equatorial guinea" OR Eritrea OR Eswatini OR Ethiopia OR gabon OR gambia OR ghana OR guinea OR "guinea-bissau" OR kenya OR lesotho OR Liberia OR Madagascar OR malawi OR mali OR Mauritania OR Mauritius OR mozambique OR namibia OR niger OR nigeria OR rwanda OR "sao tome and principe" OR "sao tome" OR Senegal OR Seychelles OR "sierra leone" OR Somalia OR Somaliland OR "South Africa" OR "South Sudan" OR sudan OR swaziland OR tanzania OR togo OR uganda OR zambia OR zimbabwe OR "sub Saharan Africa" OR "subsaharan Africa")

#15 Thesaurus: ("Sub Saharan Africa" OR "Africa South of Sahara" OR "Angola" OR "Botswana" OR "Comoros" OR "Lesotho" OR "Mozambique" OR "Namibia" OR "South Africa" OR "Swaziland" OR "Zambia" OR "Zimbabwe" OR "Burundi" OR "Cameroon" OR "Central African Republic" OR "Chad" OR "Congo" OR "Congo Democratic Republic" OR "Equatorial Guinea" OR "Gabon" OR "Sao Tome and Principe" OR "Ethiopia" OR "Eritrea" OR "Kenya" OR "Madagascar" OR "Malawi" OR "Rwanda" OR "Seychelles" OR "Somalia" OR "South Sudan" OR "Sudan" OR "Tanzania" OR "Uganda" OR "Benin" OR "Burkina Faso" OR "Cape Verde" OR "Gambia" OR "Ghana" OR "Guinea" OR "Guinea-Bissau" OR "Liberia" OR "Mali" OR "Mauritania" OR "Niger" OR "Nigeria" OR "Senegal" OR "Sierra Leone" OR "Togo")

#16 #13 OR #14 OR #15

#17 #4 AND #8 #12 AND #16

#18 #17 AND yr:[2010 TO 2021] AND English

#19 #18 AND Document Type: Journal Article & Journal Issue

**With NARROW implementation science search strategy**

#1 Article Title: (adolescent OR adolescents OR adolescence OR teenager OR teenagers OR teen OR teens OR youth OR youths OR “young adult” OR “young adults”)

#2 Abstract: (adolescent OR adolescents OR adolescence OR teenager OR teenagers OR teen OR teens OR youth OR youths OR “young adult” OR “young adults”)

#3 Subject term/Thesaurus: "adolescents" OR "young adults"

#4 #1 OR #2 OR #3

#5 Title: ((prevention OR prevent* OR preventative OR screen* OR screening* OR treatment* OR treat* OR care OR therapy OR therapies OR therapeutic* OR “preventive health service” OR “primary prevention” OR “secondary prevention” OR “tertiary prevention” OR “prevention and control”) AND (HIV OR hiv1 OR hiv2 OR hiv-1* OR hiv-2* OR AIDS OR “human immunodeficiency virus” OR “human immunodeficiency viruses” OR “human immunedeficiency virus” OR “human immunedeficiency viruses” OR “human immuno-deficiency virus” OR “human immuno-deficiency viruses” OR “acquired immunodeficiency syndrome” OR “acquired immunodeficiency syndromes” OR “acquired immunedeficiency syndrome” OR “acquired immunedeficiency syndromes” OR “acquired immuno-deficiency syndrome” OR “acquired immuno-deficiency syndromes” OR “acquired immune-deficiency syndrome” OR “acquired immune-deficiency syndromes” OR “acquired immune deficiency syndrome”))

#6 Abstract: ((prevention OR prevent* OR preventative OR screen* OR screening* OR treatment* OR treat* OR care OR therapy OR therapies OR therapeutic* OR “preventive health service” OR “primary prevention” OR “secondary prevention” OR “tertiary prevention” OR “prevention and control”) AND (HIV OR hiv1 OR hiv2 OR hiv-1* OR hiv-2* OR AIDS OR “human immunodeficiency virus” OR “human immunodeficiency viruses” OR “human immunedeficiency virus” OR “human immunedeficiency viruses” OR “human immuno-deficiency virus” OR “human immuno-deficiency viruses” OR “acquired immunodeficiency syndrome” OR “acquired immunodeficiency syndromes” OR “acquired immunedeficiency syndrome” OR “acquired immunedeficiency syndromes” OR “acquired immuno-deficiency syndrome” OR “acquired immuno-deficiency syndromes” OR “acquired immune-deficiency syndrome” OR “acquired immune-deficiency syndromes” OR “acquired immune deficiency syndrome”))

#7 Thesaurus: (("HIV" OR "human immunodeficiency viruses" OR "HIV-1" OR "HIV-1 infections" OR "HIV-2" OR "HIV-2 infections" OR "HIV-I" OR "HIV-II" OR "HIV infections" OR "AIDS" OR "acquired immune deficiency syndrome" OR "AIDS HTLV" OR "AIDS HTLV-I" OR "AIDS HTLV-II") AND ("prevention" OR "screening" OR "therapeutics" OR "therapy" OR "treatment"))

#8 #5 OR #6 OR #7

#9 Title: (implementing OR implementation OR “implementation theory” OR “implementation theories” OR "implementation science" OR "implementation research" OR “implementation intervention” OR “implementation interventions” OR "implementation strategy" OR "implementation strategies" OR “implementation framework” OR “implementation frameworks” OR “implementation barrier” OR “implementation barriers” OR “intervention research” OR “intervention oriented research” OR “improvement science” OR "dissemination research" OR "dissemination science" OR "translational science" OR "diffusion of innovation" OR "impact evaluation" OR “impact evaluations” OR “impact research” OR “knowledge transfer” OR “transfer knowledge” OR “knowledge exchange” OR “knowledge translation” OR “knowledge to action” OR “research utilization” OR (“organizational readiness” AND change) OR "ecological framework" OR "ecological frameworks" OR "Re-AIM" OR "reach effectiveness adoption implementation maintenance" OR PARIHS OR "promoting action on research implementation in health services" OR CFIR OR "PRECEDE PROCEED" OR “interactive systems framework” OR “interactive systems frameworks” OR AIF OR “Active Implementation Framework” OR “Active Implementation Frameworks” OR “Consolidated Framework for Implementation Research” OR NICS OR PRISM OR SURE OR TDF OR “Theoretical Domains Framework” OR “Theoretical Domains Frameworks” OR TICD OR “Exploration Preparation Implementation Sustainment framework” OR EPIS OR "Health Plan Implementation”)

#10 Abstract: (implementing OR implementation OR “implementation theory” OR “implementation theories” OR "implementation science" OR "implementation research" OR “implementation intervention” OR “implementation interventions” OR "implementation strategy" OR "implementation strategies" OR “implementation framework” OR “implementation frameworks” OR “implementation barrier” OR “implementation barriers” OR “intervention research” OR “intervention oriented research” OR “improvement science” OR "dissemination research" OR "dissemination science" OR "translational science" OR "diffusion of innovation" OR "impact evaluation" OR “impact evaluations” OR “impact research” OR “knowledge transfer” OR “transfer knowledge” OR “knowledge exchange” OR “knowledge translation” OR “knowledge to action” OR “research utilization” OR (“organizational readiness” AND change) OR "ecological framework" OR "ecological frameworks" OR "Re-AIM" OR "reach effectiveness adoption implementation maintenance" OR PARIHS OR "promoting action on research implementation in health services" OR CFIR OR "PRECEDE PROCEED" OR “interactive systems framework” OR “Theoretical Domains Framework” OR “interactive systems frameworks” OR “Theoretical Domains Frameworks” OR AIF OR “Active Implementation Framework” OR “Active Implementation Frameworks” OR “Consolidated Framework for Implementation Research” OR NICS OR PRISM OR SURE OR TDF OR “Theoretical Domains Framework” OR “Theoretical Domains Frameworks” OR TICD OR “Exploration Preparation Implementation Sustainment framework” OR EPIS OR "Health Plan Implementation”)

#11 Thesaurus: ("implementation" OR "implementation of research" OR "diffusion of research" OR "project implementation")

#12 #10 OR #11 OR #12

#13 Title: (angola OR benin OR botswana OR "burkina faso" OR burundi OR "cabo verde" OR cameroon OR "central African republic" OR chad OR comoros OR "Cote d Ivoire" OR "Ivory Coast" OR "Democratic Republic of the Congo" OR congo OR "republic of the congo" OR "equatorial guinea" OR Eritrea OR Eswatini OR Ethiopia OR gabon OR gambia OR ghana OR guinea OR "guinea-bissau" OR kenya OR lesotho OR Liberia OR Madagascar OR malawi OR mali OR Mauritania OR Mauritius OR mozambique OR namibia OR niger OR nigeria OR rwanda OR "sao tome and principe" OR "sao tome" OR Senegal OR Seychelles OR "sierra leone" OR Somalia OR Somaliland OR "South Africa" OR "South Sudan" OR sudan OR swaziland OR tanzania OR togo OR uganda OR zambia OR zimbabwe OR "sub Saharan Africa" OR "subsaharan Africa")

#14 Abstract: (angola OR benin OR botswana OR "burkina faso" OR burundi OR "cabo verde" OR cameroon OR "central African republic" OR chad OR comoros OR "Cote d Ivoire" OR "Ivory Coast" OR "Democratic Republic of the Congo" OR congo OR "republic of the congo" OR "equatorial guinea" OR Eritrea OR Eswatini OR Ethiopia OR gabon OR gambia OR ghana OR guinea OR "guinea-bissau" OR kenya OR lesotho OR Liberia OR Madagascar OR malawi OR mali OR Mauritania OR Mauritius OR mozambique OR namibia OR niger OR nigeria OR rwanda OR "sao tome and principe" OR "sao tome" OR Senegal OR Seychelles OR "sierra leone" OR Somalia OR Somaliland OR "South Africa" OR "South Sudan" OR sudan OR swaziland OR tanzania OR togo OR uganda OR zambia OR zimbabwe OR "sub Saharan Africa" OR "subsaharan Africa")

#15 Thesaurus: ("Sub Saharan Africa" OR "Africa South of Sahara" OR "Angola" OR "Botswana" OR "Comoros" OR "Lesotho" OR "Mozambique" OR "Namibia" OR "South Africa" OR "Swaziland" OR "Zambia" OR "Zimbabwe" OR "Burundi" OR "Cameroon" OR "Central African Republic" OR "Chad" OR "Congo" OR "Congo Democratic Republic" OR "Equatorial Guinea" OR "Gabon" OR "Sao Tome and Principe" OR "Ethiopia" OR "Eritrea" OR "Kenya" OR "Madagascar" OR "Malawi" OR "Rwanda" OR "Seychelles" OR "Somalia" OR "South Sudan" OR "Sudan" OR "Tanzania" OR "Uganda" OR "Benin" OR "Burkina Faso" OR "Cape Verde" OR "Gambia" OR "Ghana" OR "Guinea" OR "Guinea-Bissau" OR "Liberia" OR "Mali" OR "Mauritania" OR "Niger" OR "Nigeria" OR "Senegal" OR "Sierra Leone" OR "Togo")

#16 #13 OR #14 OR #15

#17 #4 AND #8 AND #12 AND #16

#18 #17 AND yr:[2010 TO 2021] AND English

#19 #18 AND Document Type: Journal Article & Journal Issue
