# Supplementary material for: Implementation Science for the Prevention and Treatment of HIV among Adolescents and Young Adults in Sub-Saharan Africa: A Scoping Review
Source: AIDS Behav. 2022 Aug 10;27(Suppl 1):7–23. doi: 10.1007/s10461-022-03770-x (PMC10191963; doi:10.1007/s10461-022-03770-x)
Supplement: Supplementary file 2 — Supplementary Material 2 [file 10461_2022_3770_MOESM2_ESM.docx]

**Supplement File 1. AHISA Scoping Review Code Book**

**Primary question**: What is the nature of IS research for adolescent HIV in SSA?

**Secondary questions**:

- What is the volume of IS research published in the African region on adolescent and HIV?
- Where are published studies coming from (e.g., country, authors)?
- What phase of implementation do the studies address?
- What IS frameworks, theories, and/or models were used in the studies?
- What implementation strategies were used?
- What implementation outcomes were assessed (e.g., acceptability, adoption, affordability, appropriateness)?
- What areas of HIV were addressed (e.g., prevention, treatment)?
- What type of intervention were tested (e.g., peer counseling, PrEP)?
- Where did the study interventions take place (e.g., school, community clinic)?
- How was the intervention delivered (e.g., tech, face-to-face, social media)?
- What study designs were used (e.g., IS type, hybrid, experimental, observational)?
- What were the study populations (e.g., HIV status, age group)?
- Who delivered the intervention (i.e., practitioner population)?

**Coding Questions^[[1]](#footnote-1)^**

1. Rater Name
2. Article number (from Covidence)
3. Lead Author Last Name
4. Publication Year
5. Should article be excluded? If so, why?
6. Article Title, Journal name, other article identifiers
7. In what country is the study set?
8. Age range of the population: ____
9. What is the gender of the study population for the intervention/EBP?
   1. Male
   2. Female
   3. Both male and female
   4. Transgender
   5. All of the above
   6. Not specified
10. What were the study populations HIV status?
    1. Positive
    2. Negative
    3. Both
    4. Unknown
    5. Not specified
    6. All of the above
11. What was the HIV continuum of care focus of the study? Select all that apply.
    1. Prevention
    2. Diagnosis
    3. Link to Care/ Receiving Treatment
    4. Retained in Care
    5. Viral Suppression
    6. All of the above
12. What was the intervention?
13. How is the intervention being delivered? Select all that apply.
    1. Social marketing
    2. Mobile technology/Apps
    3. Online
    4. Face-to-face
    5. Other- include in the notes
    6. Not stated
    7. Not applicable (no intervention is being delivered)
14. What implementation outcomes are assessed? Select all that apply.
    1. None
    2. Acceptability
    3. Adoption
    4. Appropriateness
    5. Costs
    6. Feasibility
    7. Fidelity
    8. Penetration
    9. Sustainability
    10. Scale-up
    11. Other- include in the notes
15. What implementation strategy or strategies based on the Waltz 2015 [article](https://www.ncbi.nlm.nih.gov/pmc/articles/PMC4527340/) is/are being used, if any? Select all that apply.
    1. Engage consumers
    2. Use evaluative and iterative strategies
    3. Change infrastructure
    4. Adapt and tailor to the context
    5. Develop stakeholder interrelationships
    6. Utilize financial strategies
    7. Support clinicians
    8. Provide interactive assistance
    9. Train and educate stakeholders
    10. None
16. What phase(s) of implementation research does the study address? Select all that apply.
    1. Pre-implementation (e.g., efficacy, effectiveness and/or cost-effectiveness of an intervention; adapting an intervention)
    2. Hybrid Type 1: (1°) Test effectiveness, (2°) collect data on implementation process (barriers/facilitators)
    3. Hybrid Type 2: (1°a) Test effectiveness and (1°b) test implementation
    4. Hybrid Type 3: (1°) Test implementation and (2°) evaluate effectiveness
    5. Describe implementation process (e.g., barriers/facilitators but excluding hybrid designs)
    6. Test implementation strategy/strategies
    7. Sustainability (e.g., describe sustainability; interventions to support/enhance sustainability)
    8. Measurement Development
    9. Not implementation
    10. Other, please specify:
17. What implementation model, theory or framework is being employed in the study? Select all that apply.
    1. None
    2. Consolidated Framework for Implementation Research
    3. RE-AIM (Reach, Effectiveness-Adoption, Implementation, Maintenance)
    4. Diffusion of Innovations
    5. EPIS (Exploration, Preparation, Implementation, Sustainment)
    6. Interactive Systems Framework
    7. Theory of Organizational Readiness for Change (Weiner’s Model)
    8. Other, please specify:
18. What type of study design is employed?
    1. Experimental [Manipulation with randomization (e.g., RCT, pragmatic RCT, dynamic wait-listed control, cluster RCT)]
    2. Quasi-experimental [Manipulation but no randomization (e.g., interrupted time series, non-equivalent control group)]
    3. Observational (e.g., cross-sectional, descriptive)
    4. Pre-post design
    5. Modeling (e.g., Agent Based Modeling, Systems Modeling)
    6. Case Study
    7. Social network analysis
    8. Mixed methods
    9. Other, please specify:
19. Where are the first and last author’s institutions?
20. To what extent were youth engaged in the study? (Select best option):
    1. No youth engagement - the absence of participatory approaches or activities during research.
    2. Minimal youth engagement - youth being consulted to get their opinions, assigned specific roles, or informed about events surrounding research activities, without any decision-making power.
    3. Moderate youth engagement - adult-initiated activities with shared decision making between youth and adults.
    4. Substantial youth engagement - research activities that were youth-initiated and directed
    5. Not applicable

1. See Appendix A for a list of how these coded items respond to the secondary questions. [↑](#footnote-ref-1)
